# The role of macrophages in aortic physiology and pathophysiology

**DOI:** 10.3389/fimmu.2026.1889403

**Published:** 2026-07-30

**Authors:** Yu-Xuan Zhang, Li-qing Jiang, Yong-Cang Tian, Chao Xue, Ben-Jian Bai, Wei-Xun Duan, Xiao Du, De-Jun Liu, Hong-Tao Xie, Tai-Bao Nie, Zhuang-Yi Wei

**Affiliations:** 1 Shaanxi University of Chinese Medicine, Xianyang, Shaanxi, China; 2 Department of Cardiovascular Surgery, The First Affiliated Hospital, The Air Force Medical University, Xi’an, Shaanxi, China; 3 Department of Cardiovascular Surgery, The Second Affiliated Hospital of Shaanxi University of Chinese Medicine Xi’an New Area Central Hospital, Xianyang, China

**Keywords:** aorta, aortic disease, inflammation, macrophage polarization, macrophages

## Abstract

The aorta is one of the major arterial vessels in the human body, serving as the principal conduit for the high-velocity blood ejected from the heart. For a long time, its function was largely understood in this limited hemodynamic context. However, with the advancement of research, its roles in metabolism, endocrine regulation, neurovascular modulation, and immune responses to stress have been increasingly recognized. Notably, the 2024 guidelines jointly issued by the European Association for Cardio-Thoracic Surgery (EACTS) and the Society of Thoracic Surgeons (STS) have, for the first time, formally proposed that the aorta should be regarded as an “independent organ.” This conceptual paradigm shift marks a new stage in the understanding of aortic biology and function. Macrophages are key components of the innate immune system and have attracted extensive attention due to their remarkable heterogeneity across diverse physiological and pathological conditions. In recent years, multiple macrophage subsets have been identified within the Aortic Physiology where they have been shown to play essential roles not only in vascular homeostasis but also in a wide spectrum of disease-related processes. This review systematically summarizes the roles of macrophages in both the physiological and pathophysiological contexts of the aorta, with the aim of providing new insights and potential directions for future research, as well as novel perspectives for the diagnosis and treatment of aortic diseases.

## 1 Introduction

### 1.1 Physiological functions and heterogeneity of aortic macrophages

Aortic diseases primarily include atherosclerosis, abdominal aortic aneurysm (AAA), and aortic dissection (AD), all of which represent major causes of cardiovascular-related mortality. Despite their distinct clinical manifestations, these conditions share common pathophysiological features, including chronic low-grade inflammation, progressive degradation of the vascular wall, and dysregulation of the local immune microenvironment (1). In recent years, accumulating evidence has demonstrated that macrophages are not only among the predominant immune cell populations within the inflamed aorta but also serve as central regulatory hubs linking multiple key pathological processes, including lipid metabolism dysregulation, extracellular matrix remodeling, vascular smooth muscle cell injury, and immune homeostasis imbalance (2, 3).

The second population comprises tissue-resident macrophages (RTMs), which are primarily derived from embryonic yolk sac and fetal liver progenitors. These cells are maintained long-term through local self-renewal and contribute to vascular homeostasis by clearing apoptotic cells, preserving immune equilibrium within the vessel wall, and modulating the adventitial microenvironment (9). Recent studies have further demonstrated that Lyve1+ RTMs can secrete Sparcl1 to inhibit aberrant lymphangiogenesis and tertiary lymphoid structure formation, thereby attenuating AAA progression and highlighting their vasculoprotective functions (10).

The third population consists of macrophages associated with the intima of the aorta (MacAIR), which are predominantly localized in regions of disturbed blood flow. These cells are characterized by the expression of signature markers including Mmp12, F11r, and Asb2. They participate in fibrin clearance, regulation of coagulation-related processes, and early foam cell formation, thereby playing an important role in the initiation of atherosclerosis (11).

### 1.2 Ontogeny and disease-associated functions of aortic macrophages

The traditional view holds that aortic macrophages are primarily derived from circulating monocytes, which are recruited to the vascular wall under inflammatory stimuli and subsequently differentiate into mature macrophages upon tissue infiltration (5). However, with the advent of single-cell RNA sequencing (scRNA-seq), lineage-tracing approaches, and spatial transcriptomics, it has become increasingly evident that macrophage populations within the aorta exhibit marked heterogeneity in both ontogeny and functional specialization, with evidence of distinct developmental trajectories and division of labor across subsets (6, 7).

Current evidence suggests that at least three major macrophage populations exist within the aorta. The first population consists of monocyte-derived macrophages (Mo-Macs), which originate predominantly from bone marrow–derived Ly6Chi monocytes. These cells are robustly recruited to the aortic wall under conditions of inflammation or tissue injury, where they exhibit strong pro-inflammatory, chemotactic, and matrix-degrading properties. As such, they serve as key effector cells driving atherosclerotic plaque progression and AAA expansion (5, 8).

In atherosclerosis, endothelial injury and dysfunction increase vascular permeability, allowing the retention of modified low-density lipoproteins (mLDL) within the arterial intima and promoting the recruitment of circulating monocytes. After infiltrating the intima and differentiating into macrophages, these cells internalize large amounts of mLDL, leading to foam cell formation, amplification of intraplaque inflammation, and eventual necrotic core development. In abdominal aortic aneurysm and aortic dissection, macrophages contribute to disease progression by secreting a range of pro-inflammatory cytokines, matrix metalloproteinases (MMPs), and reactive oxygen species (ROS), which collectively drive elastic fiber degradation, vascular wall thinning, and adverse aortic remodeling (4). Collectively, macrophages have emerged as key cellular mediators in the immune-related pathogenesis of aortic diseases and are increasingly recognized as important targets for understanding disease mechanisms and developing potential therapeutic interventions.

Beyond ontogenetic heterogeneity, aortic macrophages also exhibit pronounced functional plasticity and polarization dynamics. In response to inflammatory stimuli, aging, mechanical stress, and metabolic perturbations, distinct macrophage subsets can undergo dynamic polarization between pro-inflammatory M1-like and anti-inflammatory M2-like states, while also displaying a continuum of intermediate phenotypes rather than discrete categories. These functional states are orchestrated through coordinated regulation of multiple signaling pathways, including NF-κB, JAK/STAT, NLRP3 inflammasome activation, which promotes the maturation of IL-1β and IL-18, and TGF-β signaling, thereby contributing to inflammatory amplification, extracellular matrix degradation, and vascular remodeling (2, 12, 13).

Recent single-cell transcriptomic studies have further elucidated the temporal and spatial evolution of aortic macrophage populations across different stages of disease progression, revealing substantial subset remodeling and functional reprogramming. These findings provide new conceptual insights into the immune-pathological mechanisms underlying aortic diseases (6, 7). Collectively, a systematic characterization of the developmental origins, functional properties, and disease-associated roles of heterogeneous aortic macrophage populations is essential for identifying novel immunomodulatory strategies and potential precision therapeutic targets in aortic disease.

## 2 Ontogeny and functional diversity of aortic macrophages

**Figure 1:**
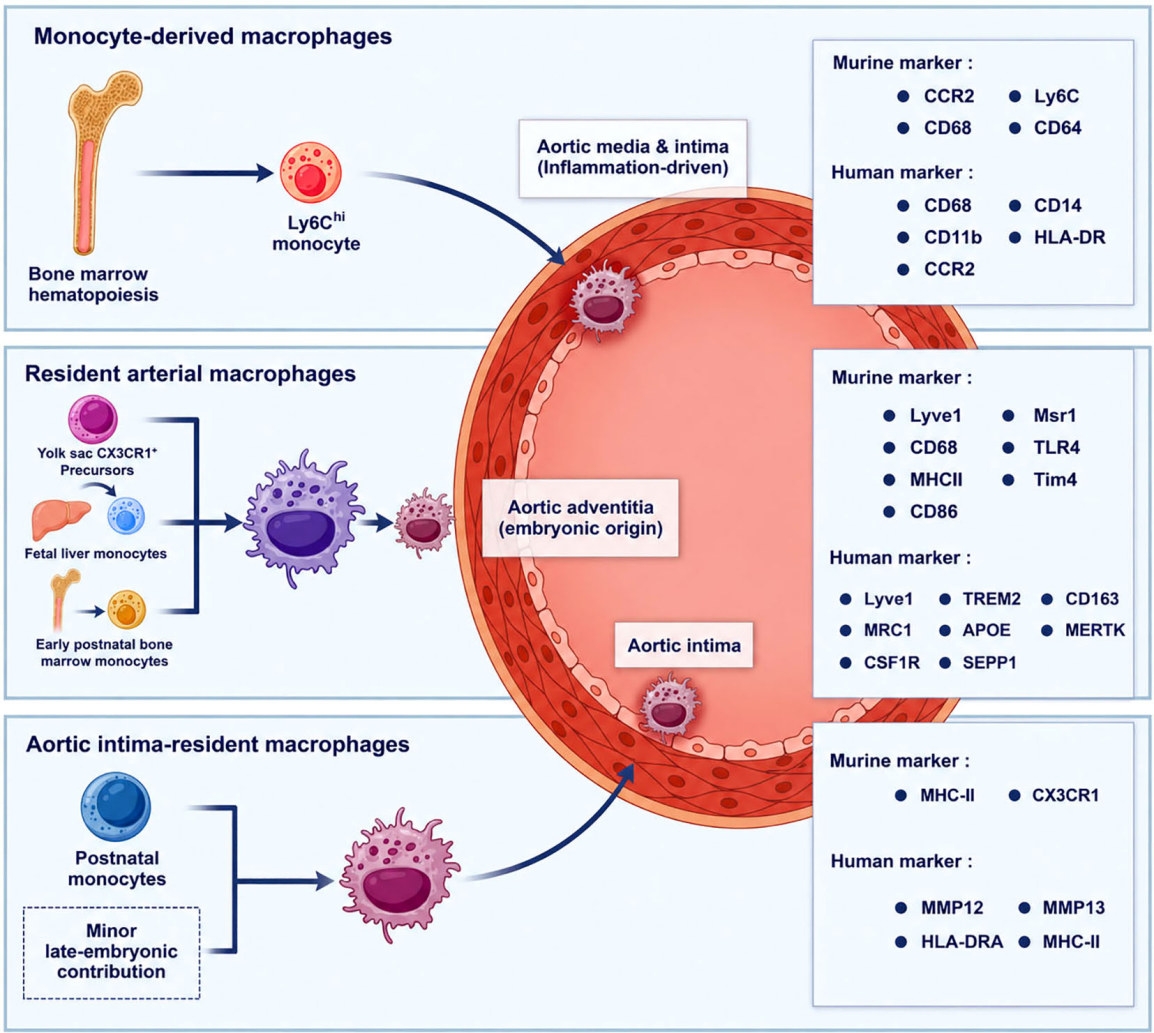
Developmental origins, anatomical distribution, and representative markers of major macrophage populations in the aorta. Schematic overview of the three principal macrophage populations identified in the aortic wall. Monocyte-derived macrophages (Mo-Macs) originate predominantly from bone marrow hematopoiesis and are continuously replenished by circulating Ly6C^hi^ monocytes under inflammatory conditions. These cells preferentially accumulate within the aortic intima and media and are characterized by strong pro-inflammatory and matrix-remodeling activities. Resident arterial macrophages (RTMs) are derived mainly from embryonic yolk sac progenitors, fetal liver monocytes, and a minor contribution of early postnatal bone marrow monocytes. RTMs are maintained through local self-renewal and are primarily located within the adventitia, where they contribute to immune surveillance, apoptotic cell clearance, and maintenance of vascular homeostasis. Aortic intima-resident macrophages (MacAIR) arise predominantly from postnatal monocytes with a limited late-embryonic contribution and reside within the intimal layer, where they participate in fibrin clearance, coagulation-related regulation, and early atherogenic responses. Representative murine and human markers reported for each macrophage population are summarized in the corresponding panels. Figure created by the authors using BioRender based on published studies summarized in this review.

Macrophages are established during embryonic development and constitute a central component of the innate immune system, playing indispensable roles in maintaining tissue homeostasis and normal organ function through processes such as phagocytosis and efferocytosis across nearly all physiological systems (14). Based on their developmental origin, macrophages can generally be classified into two major categories as shown in **Figure 1**. The first category comprises mo-macs, which are continuously replenished by bone marrow hematopoietic stem cells (HSCs) (15). This population represents the earliest recognized macrophage subset and is traditionally associated with tissue clearance of apoptotic cells, cellular debris, and damaged tissue components, as well as nutrient acquisition and host defense against invading pathogens (16). In the context of aortic disease, mo-macs are generally considered to arise from circulating monocytes that infiltrate the vascular wall in response to local inflammation or tissue injury and subsequently differentiate within the affected tissue (17). CD68, CCR2, HLA-DR, CD14, and CD11b are key characteristics of monocyte-derived macrophages in human aortic tissue (18). In murine models, the classical inflammatory monocyte-derived phenotype is typically characterized as Ly6ChiCCR2+CD11b+. Among these markers, Ly6C and CCR2 are widely recognized as key indicators of the recruitment potential of bone marrow–derived inflammatory monocytes, whereas CX3CR1 and CD115 (CSF1R) participate in monocyte migration, survival, and tissue infiltration. Following tissue entry, these cells progressively acquire features of mature macrophages, including expression of F4/80 (Adgre1), CD64 (Fcgr1), MerTK, and CD68 (15). Mouse monocyte-derived macrophages Mo-Macs show marked transcriptional heterogeneity. Inflammatory Mo-Macs represent a distinct subset. They highly express classical inflammation-associated markers, including CCR2, Ly6C, CD64, and CD68 (19). These markers are commonly used to define their inflammatory phenotype. These populations are particularly enriched in models of AAA, AD, infection, and tissue injury. In contrast, during the tissue repair phase, subsets of Mo-Macs gradually upregulate Arg1and Mrc1 (CD206) reflecting a transition toward reparative and anti-inflammatory phenotypes (18). The second major category consists of RTMs, which predominantly originate from embryonic yolk sac progenitors. Most tissue macrophage populations in both mice and humans are established during prenatal development and are maintained independently of adult hemopoiesis under steady-state conditions (20, 21). For example, microglia in the brain arise directly from primitive yolk sac–derived macrophage progenitors, alveolar macrophages (AMs) develop primarily from fetal liver monocytes, and Kupffer cells (KCs) in the liver originate from erythromyeloid progenitors (20, 21). Upon entering specific tissue microenvironments, these RTMs maintain population stability through local self-renewal and proliferation and, under homeostatic conditions, do not rely on continuous replenishment from circulating bone marrow–derived cells (20).

Within the cardiovascular system, RTMs and macrophage progenitor cells in the aorta are predominantly localized within the vascular adventitia (21). Current single-cell studies of human arterial and cardiovascular tissues have identified several representative molecular markers of resident arterial macrophages, including Lyve1 (lymphatic vessel endothelial hyaluronan receptor 1) (22), CD163 (23), MRC1 (CD206) (24), MERTK (22), CSF1R (23), APOE (22), SEPP1 (23), and TREM2 (22). In murine models, resident arterial macrophages are commonly characterized by expression of Lyve1, CD68, major histocompatibility complex class II (MHCII), CD86, the scavenger receptor Msr1, Toll-like receptor 4 (TLR4), and T-cell immunoglobulin and mucin domain-containing protein 4 (Tim4) (25). Recent single-cell studies have further demonstrated that subsets of monocyte-derived macrophages may progressively acquire features resembling resident macrophages after entering tissues. For instance, increased expression of Timd4, Lyve1, and Folr2 suggests differentiation toward a tissue-resident macrophage phenotype (15). Aortic macrophages derived from CX3CR1^+^ progenitors exhibit a dual developmental origin, including both embryonic-derived populations from the yolk sac and fetal liver, as well as perinatal populations originating from bone marrow–derived monocytes (26). Notably, fetal macrophage development precedes the emergence of hematopoietic stem cells (HSCs) (27). During early embryogenesis, primitive hematopoiesis occurs at approximately embryonic day 7.0 (E7.0), during which the posterior plate mesoderm of yolk sac blood islands generates early macrophage populations (28). At E8.5, yolk sac–derived erythromyeloid progenitors (EMPs) migrate through the circulation to the fetal liver, where they subsequently undergo expansion and differentiation (29). CX3CR1^+^ lineage-tracing studies have demonstrated that approximately 40% of embryonic aortic macrophages at E16.5 originate from yolk sac–derived EMPs and display an F4/80^hi^CD11b^low^ phenotype, with their development occurring independently of a monocyte intermediate stage (27, 30). Following E10.5, the fetal liver becomes the principal site for EMP expansion and further differentiation (29). From E16.5 until birth, a subset of macrophages arises from fetal liver monocytes in a c-Myb–dependent manner and exhibits an F4/80^int^CD11b^hi^ phenotype, accounting for approximately 28% of neonatal aortic macrophages (30). In murine models, bone marrow–derived monocytes are rapidly recruited after birth and subsequently differentiate into macrophages, accompanied by increased expression of CCL2 and adhesion molecules, including VCAM-1, E-selectin, and ICAM-1, within the aortic wall. Monocytes seeded during this developmental window progressively differentiate into adult aortic macrophages and, together with embryonically derived macrophages, establish a mixed macrophage population. However, within the first two postnatal weeks, aortic macrophages transition from the embryonic F4/80^hi^CD11b^lo^ phenotype toward an F4/80^int^CD11b^hi^ phenotype, accompanied by upregulation of MHCII expression and acquisition of functional maturity (26). In adulthood, murine aortic macrophages maintain tissue homeostasis primarily through CX3CR1–CX3CL1 axis–dependent self-renewal and local microenvironmental signaling (26), a developmental paradigm distinct from tissues such as the intestine, which rely on continuous replenishment by circulating monocytes (31). Functionally, these RTMs contribute to local homeostasis through the clearance of damaged cells and foreign materials (20).

At present, functional studies of aortic RTMs remain relatively limited. Existing evidence suggests that these cells contribute to the maintenance of aortic extracellular matrix homeostasis and arterial stiffness through interactions with hyaluronan receptors expressed on vascular smooth muscle cells (VSMCs). In particular, RTMs can secrete matrix metalloproteinase-9 (MMP-9) to degrade pericellular collagen surrounding smooth muscle cells, thereby regulating vascular matrix turnover and biomechanical properties. Consistent with this, deficiency of Lyve1^+^ macrophages has been shown to induce arterial stiffness and collagen deposition (32). In addition to the two major macrophage populations described above, the aorta also contains a specialized subset of RTMs termed macrophages associated with the intima of the aorta (Mac^AIR^) as shown in **Figure 1**. These cells were initially classified as vascular dendritic cells (vascular DCs) because they express classical dendritic cell markers, including CD11c and MHC-II. However, subsequent studies demonstrated that they do not express dendritic cell–restricted genes, including Zbtb46, Xcr1, Mycl, or Ccr7, supporting the notion that they represent a distinct macrophage population rather than bona fide dendritic cells. Single-cell transcriptomic analyses combined with reference database comparisons further confirmed the heterogeneity and unique transcriptional identity of these cells, with representative molecular markers including MMP12, F11r, Asb2, and Gngt2. Similar to other resident macrophage populations within the aorta, maintenance and survival of Mac^AIR^ critically depend on colony-stimulating factor 1 (CSF1) signaling. Although a small proportion of Mac^AIR^ develops during late embryogenesis, the majority are derived from monocytes that infiltrate the aortic intima shortly after birth. This postnatal recruitment is thought to be associated with hemodynamic and microenvironmental changes occurring after birth, including increased cardiac output, elevated arterial pressure, smooth muscle cell expansion, and extracellular matrix remodeling. Following infiltration, these monocytes progressively differentiate into Mac^AIR^ and subsequently maintain local homeostasis through self-renewal (11). Functionally, Mac^AIR^ are believed to participate in the earliest stages of foam cell formation during atherogenesis. In addition, they have been shown to play critical roles in regulating thrombin activity and clearing fibrin deposition in regions exposed to disturbed blood flow (16). Representative markers of human aortic intima-resident macrophages include MMP12, MMP13, and HLA-DRA/MHC-II–related molecules (16), whereas in murine models these cells are commonly characterized by expression of MHC-II and CX3CR1 (11, 16). Overall, current knowledge regarding macrophage function in the aorta remains incomplete, particularly with respect to RTMs and intima-associated macrophage populations, for which mechanistic studies remain scarce. Nevertheless, with the continued development of single-cell and spatial multi-omics technologies, the biological functions and pathological significance of these macrophage subsets are likely to be further elucidated in the future.

Recent studies in zebrafish, using high-resolution spatiotemporal live imaging in embryos, have identified a distinct population of macrophages residing within blood vessels, termed blood vessel-resident macrophages (bMΦs), with conserved features also observed in mice. These cells patrol the bloodstream, clear foreign particles and damaged or unfit cells, and act as first responders to endothelial injury. Notably, bMΦs emerge directly from axial blood vessels via an atypical endothelial-to-macrophage transition that is independent of Runx1 and Csf1r, representing a non-classical ontogenetic pathway distinct from canonical hematopoiesis (33). However, the existence of a fully equivalent macrophage population in mammals remains to be definitively established. With continued advances in single-cell, spatial, and *in vivo* imaging technologies, it is anticipated that additional functionally specialized macrophage subsets with diverse developmental origins will be identified. Moreover, elucidating the complex interactions among macrophage populations, as well as between macrophages and other vascular cell types, will likely provide deeper insight into the regulation of aortic homeostasis and the pathogenesis of vascular diseases.

## 3 The role of macrophages in atherosclerosis

Atherosclerosis is a chronic inflammatory disease of the arterial wall (33), predominantly affecting medium- and large-sized arteries (34). Its pathophysiological progression involves not only lipid deposition but is also critically driven by inflammatory responses (1). Macrophages play a central role in atherosclerosis, participating in multiple key processes, including foam cell formation, regulation of inflammatory responses, maintenance of plaque stability, and thrombosis development.

### 3.1 Initiation of atherosclerosis and plaque formation driven by circulating monocyte-derived macrophages

Evidence has demonstrated that all three major classes of macrophages participate in the initiation and progression of atherosclerosis, among which circulating monocyte-derived macrophages represent the most extensively studied population. Under pathological stimuli such as dyslipidemia, hypertension, and inflammatory mediators, arterial endothelial cells are among the first to upregulate adhesion molecules, accompanied by increased endothelial permeability, which facilitates the infiltration and retention of low-density lipoprotein (LDL) particles within the intimal layer of the arterial wall, where they may undergo oxidative modification. Endothelial activation promotes the recruitment of circulating monocytes to lesion sites. Once within the intima, oxidized lipoproteins are engulfed by macrophages, leading to excessive intracellular cholesterol accumulation, foam cell formation, and ultimately contributing to necrotic core development (35). In response to macrophage colony-stimulating factor (M-CSF) and other inflammatory cues, foam cells upregulate multiple scavenger receptors, including SR-A1, CD36, and LOX-1, which bind oxidized low-density lipoprotein (oxLDL) and mediate its uptake, thereby further exacerbating cholesterol accumulation (36, 37). A large number of foam cells, together with inflammatory cells, constitute the cellular core of atherosclerotic plaques (36). In the early stages of lesion development, macrophages clear lipoproteins via scavenger receptor–mediated uptake; however, this process lacks effective negative feedback regulation. Under conditions of dysregulated lipid metabolism, macrophages undergo phenotypic reprogramming, accompanied by impaired immunoregulatory function. During plaque progression, macrophages continuously secrete chemokines, cytokines, reactive oxygen and nitrogen species (ROS/RNS), and matrix-degrading proteases, thereby amplifying local inflammatory responses. Ultimately, apoptotic or necrotic macrophages release lipids and tissue factor, contributing to plaque destabilization, rupture, and subsequent thrombosis. Circulating monocytes serve as precursors of macrophages. In addition to the bone marrow, hematopoietic stem and progenitor cells in the spleen can also generate monocytes, which are mobilized and recruited to inflammatory sites, including atherosclerotic plaque (37). Monocytes are broadly classified into two subsets based on Ly6C expression: Ly6C^hi (high)^ and Ly6C^low^ monocytes (38). Ly6C^hi^ monocytes are short-lived, adhere to activated endothelium, and infiltrate the vascular wall. They can transport antigens to lymph nodes and differentiate into macrophages at inflammatory sites. In contrast, Ly6C^low^ monocytes are derived from Ly6C^hi^ monocytes, exhibit longer survival, participate in early infection responses, and survey endothelial integrity (39). In mouse models of hyperlipidemia, Ly6C^hi^ monocytes are expanded due to impaired conversion into Ly6C^low^ monocytes (38). The major chemokine receptor–ligand axes involved in monocyte recruitment include CCR2–CCL2, CX3CR1–CX3CL1, and CCR5–CCL5 (37). Both monocyte subsets express CX3CR1, with the highest expression observed in non-classical monocytes lacking CCR2 expression, thereby defining this population as non-classical monocytes (40). These chemokine axes exert distinct functional roles: CX3CR1 signal transduction promotes monocyte and macrophage survival (39); CCL2 primarily mediates mobilization of monocytes from the bone marrow; CCL5 and CXCL1 directly promote chemotaxis of monocytes to lesion sites; and CCL20 recruits Ly6C^hi^ monocytes via CCR6 engagement (39).

### 3.2 Macrophage polarization and its regulatory roles in inflammatory response

Macrophages exhibit remarkable plasticity and can undergo adaptive responses to microenvironmental cues, including interferons (IFNs), Toll-like receptor (TLR) ligands, interleukin-4/interleukin-13 (IL-4/IL-13), and IL-10, a process collectively referred to as macrophage polarization (41). Polarization is broadly categorized into classically activated pro-inflammatory M1 macrophages and alternatively activated anti-inflammatory/pro-repair M2 macrophages (42). M1 macrophages represent a pro-inflammatory phenotype induced by stimuli such as cholesterol crystals, IFN-γ, lipopolysaccharide (LPS), oxidized low-density lipoprotein (ox-LDL), TLR ligands, and pro-inflammatory cytokines (43, 44). A central triggering axis involves the synergistic action of IFN-γ (derived from Th1 and NK cells) and TLR ligands, which is further amplified by TNF and IL-1β (43, 45). In murine atherosclerotic lesions, macrophage-derived IL-12 and IL-18 promote IFN-γ production, thereby amplifying inflammatory responses and compromising vascular stability (46). M1 polarization contributes to sustained inflammatory signal transduction and exacerbates damage to surrounding tissues (47). In contrast, M2 macrophages are activated by IL-4 and IL-13 (48) and are characterized by the secretion of anti-inflammatory mediators, including IL-10 and TGF-β, efficient efferocytosis of apoptotic cells, and promotion of collagen synthesis and tissue repair, thereby contributing to plaque regression (48, 49). M2 macrophages also express chemokines such as CCL17, CCL22, and CCL24 (50). Beyond the canonical M1/M2 paradigm, multiple non-classical macrophage subsets have been identified within atherosclerotic lesions, including Mox macrophages induced by oxidized phospholipids (51), M4 macrophages induced by CXCL4 with pro-atherogenic properties (52), and M(Hb) and Mhem macrophages, which are predominantly localized in plaque neovascularization and hemorrhagic foci (53). Platelet-derived CXCL4 promotes monocyte adhesion to the endothelium and their subsequent transmigration into the subendothelial space, where they differentiate into M4 macrophages (52). Targeting macrophage polarization has therefore emerged as a promising therapeutic strategy for atherosclerosis.

### 3.3 Integrated signaling pathways controlling macrophage plasticity in atherosclerotic inflammation

**Figure 2:**
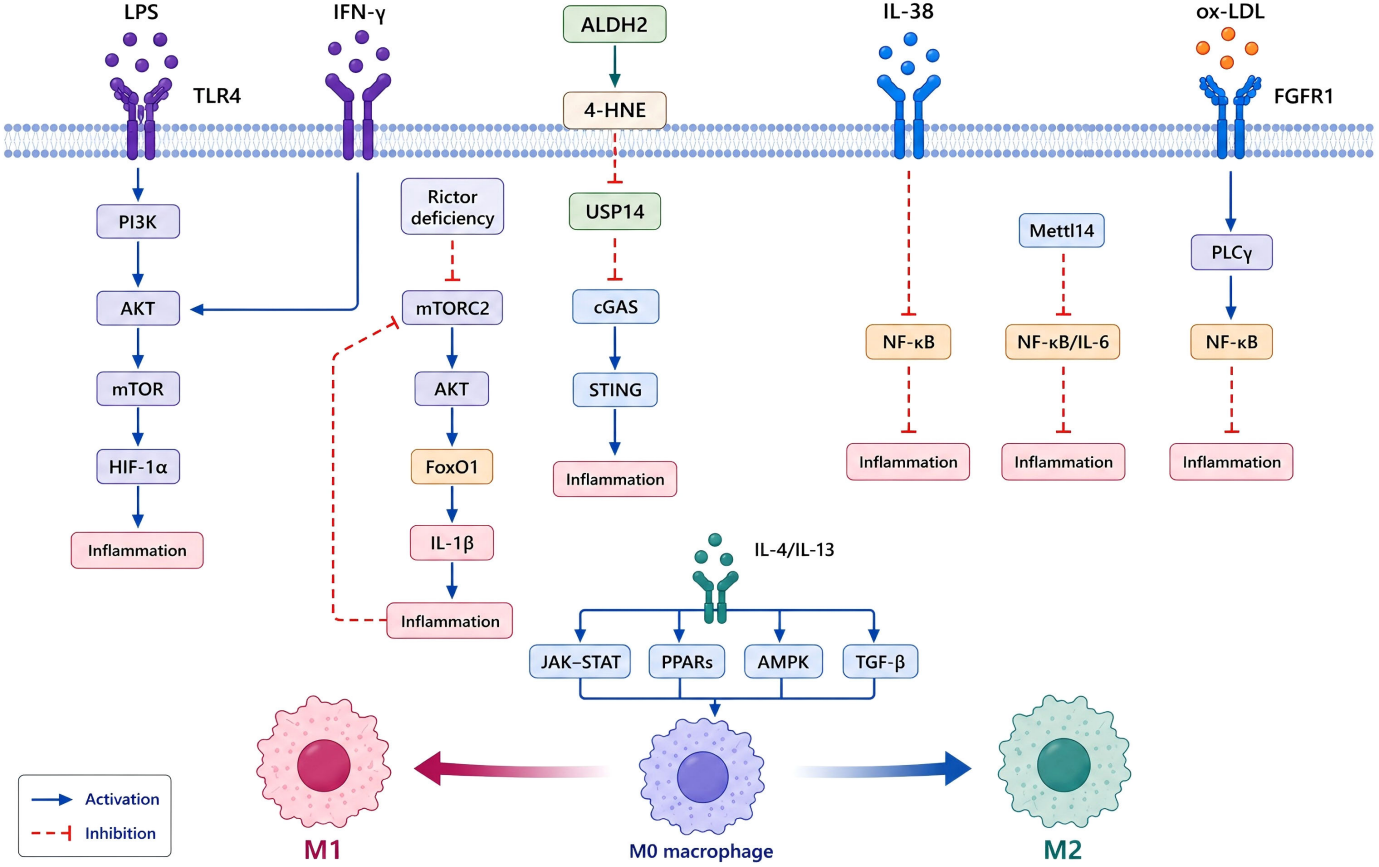
Key signaling pathways regulating macrophage polarization and inflammatory responses in aortic diseases. Lipopolysaccharide (LPS) and interferon-γ (IFN-γ) promote pro-inflammatory macrophage activation through the PI3K–AKT–mTOR–HIF-1α and mTORC2–AKT–FoxO1 signaling pathways, respectively. ALDH2 suppresses inflammation through the 4-HNE/USP14/cGAS–STING axis, whereas IL-38 inhibits NF-κB-mediated inflammatory responses. Mettl14 and oxidized low-density lipoprotein (ox-LDL) modulate macrophage inflammation through NF-κB/IL-6 and FGFR1–PLCγ–NF-κB signaling pathways, respectively. In contrast, IL-4 and IL-13 promote anti-inflammatory macrophage polarization through activation of JAK–STAT, PPARs, AMPK, and TGF-β signaling pathways. Blue solid arrows indicate activation, whereas red dashed lines indicate inhibition. The schematic illustrates the major signaling pathways involved in macrophage polarization and inflammatory regulation in aortic diseases. Created using BioRender.

The transition of macrophage polarization phenotypes is critically dependent on the regulation of intracellular signal transduction pathways as shown in **Figure 2**. In the context of atherosclerosis, multiple signal transduction cascades have been well documented. Bacterial lipopolysaccharide (LPS), as a key pro-inflammatory stimulus, drives the polarization of unpolarized (M0) macrophages toward the M1 phenotype via the PI3K–AKT–mTOR–HIF-1αsignal transduction axis, while interferon-γ (IFN-γ) further potentiates this polarization process. In contrast, IL-4 and IL-13 are the primary inducers of M2 polarization, activating one or more signal transduction pathways, including JAK–STAT, PPARs, AMPK, and TGF-β, thereby promoting the transition of M0 macrophages toward an M2 phenotype (54). The PI3K/Akt signal transduction pathway regulates macrophage survival, proliferation, migration, and polarization, thereby exerting a profound influence on the initiation and progression of atherosclerosis (AS). Activation of Akt signal transduction accelerates AS development, whereas deletion of the Rictor gene in bone marrow–derived cells disrupts the assembly of the mTORC2 complex (a key upstream regulator of Akt activation), thereby indirectly inhibiting Akt signal transduction Under these conditions, the survival and proliferative capacity of monocytes and macrophages are markedly reduced, limiting their accumulation within lesions and consequently attenuating atherosclerotic plaque formation (55). mTOR is a highly conserved serine/threonine kinase belonging to the PI3K-related kinase family and is involved in multiple pathological processes, including metabolic cardiovascular diseases. Notably, the mTORC2–Akt–FoxO1 axis constitutes a key regulatory pathway controlling macrophage IL-1β expression. mTORC2 exerts anti-atherosclerotic effects by suppressing the FoxO1–IL-1β inflammatory axis, whereas its deficiency accelerates atherosclerosis progression (56). The cGAS–STING signal transduction pathway is another critical regulator of macrophage polarization and inflammatory responses. Emerging evidence indicates that aldehyde dehydrogenase 2 (ALDH2) modulates macrophage polarization and inflammation via this pathway. ALDH2 promotes the degradation of 4-hydroxynonenal (4-HNE), inhibits cGAS de-ubiquitination, and regulates the cGAS–STING pathway through USP14, thereby suppressing macrophage polarization toward a pro-inflammatory phenotype and reducing atherosclerotic plaque formation (57). NF-κB is a central signaling pathway governing inflammatory responses. Recent studies have demonstrated that IL-38 can inhibit macrophage polarization toward the pro-inflammatory M1 phenotype by blocking this key signaling axis at an upstream level. In addition, Mettl14 has been shown to regulate macrophage-mediated inflammation in atherosclerosis via the NF-κB/IL-6 signaling pathway; knockdown of Mettl14 promotes M2 polarization of macrophages (58). Fibroblast growth factor receptor 1 (FGFR1), a member of the receptor tyrosine kinase family, is upregulated in both total protein expression and phosphorylation levels in atherosclerotic lesions and is predominantly localized in macrophages. FGFR1 promotes oxidized low-density lipoprotein (ox-LDL)-induced NF-κB–mediated inflammatory cytokine expression via PLCγ signaling, thereby facilitating plaque progression (59). In addition to these pathways, numerous other signaling cascades contribute to the development of atherosclerosis and are closely associated with macrophage function, representing important therapeutic targets for disease prevention and intervention (60). Collectively, these studies have primarily focused on circulating monocyte-derived macrophages; whether resident tissue macrophages within arterial walls possess similar signaling mechanisms remains an important unresolved question that may provide additional insights for therapeutic development.

### 3.4 Crosstalk between macrophages and the vascular immune microenvironment

The development of atherosclerosis is not driven by a single cellular component; rather, T cells also play a pivotal role in its progression. T cell–derived proteins, including versican (VCAN) and heat shock protein 90 beta family member 1 (HSP90B1), can bind to Toll-like receptors TLR1/2 and TLR2 expressed on macrophages, thereby activating pro-inflammatory macrophages, exacerbating intraplaque inflammation, and promoting plaque instability. Conversely, multiple ligands expressed on macrophages interact with receptors on CD4^+^ or CD8^+^ T cells, thereby modulating T cell proliferation and the magnitude of adaptive immune responses, ultimately forming a bidirectional feedback loop (61). In advanced stages of atherosclerosis, in addition to macrophages, VSMCs and their derived phenotypic variants constitute a major component of the necrotic core. Macrophage-derived inflammatory mediators, such as IL-1β and TNF-α, induce VSMC apoptosis and phenotypic switching. In turn, dying VSMCs release lipids and pro-inflammatory mediators that further recruit and activate macrophages, thereby establishing a self-amplifying vicious cycle that contributes to plaque progression and instability (62).

### 3.5 Programmed cell death of macrophages in the pathogenesis of atherosclerosis

In the process of atherosclerosis, in addition to macrophage polarization, multiple forms of programmed cell death, including ferroptosis, pyroptosis, and efferocytosis dysfunction, are critically involved. Ferroptosis has emerged as an important regulatory mechanism in recent years. Macrophages undergo iron overload due to increased iron uptake and dysregulated iron homeostasis. Excess ferrous iron (Fe²^+^) promotes extensive lipid peroxidation via Fenton reactions as well as enzymatic pathways involving lipoxygenases and cytochrome P450 oxidoreductases. Concurrently, the activity of the major anti-ferroptotic defense systems, including the GPX4–GSH, FSP1–CoQ10, and GCH1–BH4 pathways, is impaired, resulting in insufficient detoxification of lipid peroxides and ultimately triggering macrophage ferroptosis (63). Dying macrophages release pro-inflammatory cytokines, ROS, and MMPs, thereby exacerbating local inflammation and extracellular matrix degradation, promoting expansion of the necrotic core and thinning of the fibrous cap. In addition, iron overload further drives macrophage polarization toward a pro-inflammatory M1 phenotype, thereby amplifying inflammatory responses, accelerating foam cell formation and plaque progression, and ultimately compromising plaque stability and increasing the risk of rupture (64). Pyroptosis is also closely associated with atherosclerosis. Gasdermin D (GSDMD) and Gasdermin E (GSDME), expressed in macrophages, are key effector proteins in the pyroptotic pathway. Oxidized low-density lipoprotein (ox-LDL) can upregulate GSDME expression in macrophages and induce pyroptotic cell death (65). GSDMD-mediated pore formation in the plasma membrane is characterized by the release of inflammatory cytokines and can activate the cGAS–STING–TBK1–IRF3/NF-κB signal transduction axis, accompanied by mitochondrial membrane permeabilization and mitochondrial DNA (mtDNA) leakage, thereby exacerbating atherosclerotic plaque progression (66). Efferocytosis by macrophages refers to the process of engulfment and clearance of apoptotic cells within atherosclerotic lesions. This process reduces the release of pro-inflammatory mediators from apoptotic cells while promoting the secretion of anti-inflammatory factors by phagocytes, thereby effectively limiting necrotic core formation. However, when apoptotic cell debris accumulates over time, efferocytotic capacity becomes impaired. As a result, apoptotic cells are not efficiently cleared, leading to secondary necrosis, exacerbated inflammation, and reduced plaque stability (67).

### 3.6 Macrophage-targeted therapeutic strategies for atherosclerosis

Beyond conventional lipid-lowering therapies, increasing evidence has highlighted macrophage cell death pathways as promising therapeutic targets in atherosclerosis. MCL has been reported to delay atherosclerosis progression by selectively binding to the Arg483 residue of KEAP1, thereby inhibiting oxidized low-density lipoprotein (ox-LDL)-induced macrophage ferroptosis (68). Recent studies have demonstrated that paclitaxel (PTX) attenuates macrophage ferroptosis and improves atherosclerotic pathology via activation of the Sirt1/Nrf2/GPX4 signaling axis (69). Similarly, trihydroxyflavone (TRI), a compound with well-documented anti-inflammatory and antioxidant properties, exerts anti-atherosclerotic effects by activating the NRF2 pathway and suppressing macrophage ferroptosis, highlighting its potential as a candidate therapeutic agent (70).

In addition to small-molecule interventions, nanomedicine-based strategies have also shown therapeutic promise. IRF5 siRNA-loaded nanoparticles can selectively target specific macrophage subsets, particularly Cd11c^+^ and Trem2^+^ populations, which are efficiently internalized by lesional macrophages. Following cellular uptake, siRNA-mediated silencing of IRF5 significantly enhances macrophage efferocytosis, thereby promoting apoptotic cell clearance and reducing plaque inflammation (71). Beyond ferroptosis regulation, modulation of autophagy represents another therapeutic avenue. Arsenic trioxide (ATO) has been shown to induce reactive oxygen species (ROS) production, promote TFEB nuclear translocation, and inhibit the PI3K/AKT/mTOR signal transduction pathway, thereby enhancing macrophage autophagy and alleviating early atherosclerotic lesions. This effect has been validated in multiple cellular models, including Raw264.7, THP-1, human peripheral blood mononuclear cells, and macrophage-derived foam cells, as well as *in vivo* systems (16). Furthermore, wogonin has been reported to promote cholesterol efflux in macrophages via activation of the PPARα–KLF11–YAP1 signal transduction axis, thereby reducing lipid accumulation, suppressing foam cell formation, and ultimately attenuating plaque burden, inflammation, and instability (72).

Despite these advances, most therapeutic strategies predominantly target circulating monocyte-derived macrophages, whereas intimal-resident macrophages and RTMs, which are involved in early lesion initiation, remain insufficiently explored. Future therapeutic development should therefore pay greater attention to these macrophage subsets, enabling stage-specific intervention during atherosclerosis progression and ultimately reducing disease complications and mortality.

### 3.7 Functional roles of Mac^AIR^ and aortic tissue-resident macrophages in the pathogenesis of atherosclerosis

In addition to circulating monocyte-derived macrophages, Mac^AIR^ has been demonstrated to represent one of the earliest macrophage populations to form foam cells during atherosclerosis development. However, its proliferative capacity is limited, and as the lesion progresses, these cells are gradually replaced by infiltrating monocyte-derived macrophages (11). Consequently, relatively little is known regarding the functional roles of Mac^AIR^ in disease progression. The third major macrophage population comprises aortic RTMs, which also play a critical role in atherosclerosis. These cells are actively involved in plaque progression. In vulnerable plaque regions, hypoxia, hyperglycemia, and sustained inflammatory stimulation drive RTMs from a quiescent state toward a highly activated phenotype, accompanied by a metabolic shift resembling the Warburg effect, thereby contributing to increased plaque instability. In advanced stages of atherosclerosis, RTMs may account for up to 87% of total macrophages within plaques, and a subset of these cells may also originate from bone marrow–derived monocytes that differentiate into this population (64). Under hyperlipidemic conditions, RTMs can take up lipids and lipoproteins through passive diffusion, receptor-mediated endocytosis, and membrane transport pathways, ultimately transforming into foam cells (64). Even in late-stage disease, prominent macrophage accumulation can still be observed in the adventitial regions of atherosclerotic lesions (73). Macrophages are involved throughout the entire process of atherosclerosis initiation, progression, and plaque rupture, and their functions extend beyond classical roles in lipid uptake and foam cell formation to include inflammatory amplification, immune regulation, cell death, and tissue remodeling. With the development of single-cell RNA sequencing, lineage tracing, and spatial transcriptomics, the heterogeneity and dynamic transitions of macrophage populations from distinct origins have been increasingly elucidated. Compared with the well-characterized circulating monocyte-derived macrophages, the roles of Mac^AIR^ and aortic RTMs in early lesion initiation, local inflammatory maintenance, and plaque stability regulation remain incompletely understood. Future studies are still needed to further delineate the specific functions and regulatory mechanisms of distinct macrophage subsets across different stages of atherosclerosis, thereby providing new insights for early intervention and targeted therapeutic strategies.

## 4 The role of macrophages in abdominal aortic aneurysm

AAA is a disease characterized by localized pathological dilation of the abdominal aorta (78). The maximal aortic diameter can exceed 3 cm, and in severe cases, the degree of dilation may reach more than 50% of the normal vessel diameter (74). Rupture of the aneurysm can lead to rapid lethal hemorrhage (75). The disease is initiated by vascular injury, which subsequently triggers a cascade of inflammatory responses. Degradation of extracellular matrix (ECM) proteins by proteolytic enzymes results in progressive thinning of the arterial wall and ultimately may culminate in aortic rupture (76). In dilated abdominal aortic segments, inflammatory cell infiltration, elastin degradation, and vascular smooth muscle cell apoptosis are commonly observed, with macrophages representing the predominant inflammatory cell population (77). Atherosclerosis is an important risk factor for AAA. As a key component in the pathogenesis of atherosclerosis, macrophages also play a central role in the initiation and progression of AAA, with particular accumulation observed in the adventitial layer and luminal thrombus regions of aneurysmal lesions (77).

### 4.1 Recruitment and polarization of circulating monocyte-derived macrophages in abdominal aortic aneurysm

In the early stage of abdominal aortic aneurysm (AAA), the majority of macrophages are derived from circulating monocytes (78). Consistent with the pathophysiological processes observed in atherosclerosis, macrophages do not spontaneously accumulate at aneurysmal sites; rather, their recruitment is driven by two key signals: (i) degradation products of the ECM, which reflect structural destruction of the aortic wall, and (ii) chemokines, including MCP-1, IL-8, and TNF-α. These chemotactic factors and recruited macrophages co-localize within the same lesional regions, thereby establishing a localized pro-inflammatory microenvironment (75).

Macrophage polarization also represents a critical pathophysiological mechanism in AAA development. The polarization of macrophages toward either the pro-inflammatory M1 phenotype or the reparative M2 phenotype is highly dependent on the disease stage. In the early phase of aneurysm formation, pro-inflammatory M1 macrophages predominate and are particularly prominent during aneurysmal expansion. Following stabilization of smooth muscle cell (SMC) loss and ECM degradation, macrophages gradually shift toward a reparative M2 phenotype. In addition to monocyte-derived macrophages, these phenotypic transitions are also observed within resident macrophage populations. During the stable growth phase of aneurysms, reparative macrophages contribute to tissue homeostasis and remodeling (79). Importantly, maintenance of a balanced M1/M2 ratio is essential for preserving aortic tissue homeostasis (80).

### 4.2 Macrophage-associated signaling pathways in the pathogenesis and progression of abdominal aortic aneurysm

Multiple canonical signal transduction pathways have been identified as key regulators in the pathogenesis of AAA, including NF-κB (81), NLRP3/caspase-1/GSDMD (81), JAK2/STAT1 (82), and mTOR–AKT (83). These pathways differentially regulate macrophage polarization, migration, and inflammatory activation, thereby exerting either pro- or anti-aneurysmal effects, with extensive crosstalk observed among them. Notably, the majority of these signal transduction cascades primarily promote M1 macrophage polarization, thereby exacerbating vascular injury and inflammation. Among them, the RAGE-mediated NF-κB signal transduction pathway induces macrophage polarization toward the pro-inflammatory M1 phenotype, leading to excessive secretion of pro-inflammatory cytokines and subsequent aggravation of vascular inflammation and structural destruction (81). In addition, the transmembrane protein NINJ1 promotes macrophage recruitment to vascular inflammatory sites by competitively binding ANXA2 and activating the TLR4/NF-κB/CCR2 axis, thereby enhancing inflammatory cell infiltration and exacerbating local vascular injury (84). TRAF family proteins further mediate activation of the NLRP3/caspase-1/GSDMD signal transduction pathway, amplifying inflammatory damage and structural deterioration of the aortic wall (85). Moreover, CCL7 promotes M1 polarization of macrophages through activation of the CCR1/JAK2/STAT1 axis, thereby accelerating AAA formation and progression (86). Conversely, several signal transduction pathways have been shown to exert protective effects by promoting anti-inflammatory M2 macrophage polarization and suppressing excessive inflammatory responses, thereby preserving aortic integrity and attenuating AAA progression. The SR-A1–STAT3–IRG1 axis effectively suppresses local vascular inflammation and alleviates AAA-associated pathological damage (82). Similarly, CD74 regulates macrophage polarization balance via PKM2-mediated modulation of the TSC2–mTOR–AKT signal transduction pathway, thereby contributing to the regulation of AAA pathogenesis (83). Furthermore, crosstalk between different macrophage polarization states has also been reported. M1 and M2 macrophages can communicate via STAT1-dependent signaling, jointly contributing to the regulation of inflammatory microenvironment homeostasis in AAA (86).

### 4.3 Regulation of macrophage function by endogenous modulatory molecules

Multiple endogenous regulatory molecules can modulate macrophage polarization, inflammatory cytokine secretion, and vascular extracellular matrix metabolism, thereby contributing to the initiation and progression of AAA. These molecules exhibit bidirectional regulatory properties and represent key potential therapeutic targets in AAA. Among protective regulatory factors, low expression of the transcription factor TCF3 activates the miR-143-5p/CCL20 signal transduction axis, promoting macrophage polarization toward the M2 phenotype, suppressing aortic inflammation, and thereby inhibiting AAA progression (87). Growth differentiation factor 15 (GDF15) has been shown to activate the TGF-βR/SMAD2/3 pathway and upregulate AREG expression, effectively suppressing pro-inflammatory M1 macrophage polarization while enhancing anti-inflammatory M2 polarization, thereby alleviating aortic wall injury (88). Sirtuin 1 (SIRT1) maintains local inflammatory homeostasis in the aorta by inhibiting the NF-κB pro-inflammatory pathway and activating the STAT6 anti-inflammatory pathway, thereby suppressing AAA development (69). CD74 promotes M1 polarization through PKM2-mediated activation of the mTOR pathway; however, mesenchymal stem cell–derived exosomes (MSC-Exo) can downregulate CD74 expression, thereby reversing macrophage polarization toward an M2 phenotype and exerting anti-inflammatory effects (83). In addition, Fcγ receptor (FcγR) deficiency induces macrophage polarization toward the M2 phenotype while preserving the contractile phenotype of vascular smooth muscle cells, thereby maintaining aortic structural integrity (89). Furthermore, GSDME gene deletion increases the proportion of anti-inflammatory TIM4^+^ resident macrophages while reducing pro-inflammatory MHCII^+^ macrophages, thereby improving the inflammatory microenvironment of the aorta (65).

### 4.4 Mechanisms of macrophage injury mediated by pro-inflammatory molecules

Among injurious regulatory molecules, ADAR1 is highly and specifically expressed in M1 macrophages and can exacerbate aortic inflammation and AAA progression by promoting Drosha degradation, thereby blocking the biogenesis of anti-inflammatory microRNAs; accordingly, targeted inhibition of ADAR1 effectively alleviates AAA-associated pathological damage (90). PCSK9 has been reported to induce M1 macrophage polarization and enhance matrix metalloproteinase-9(MMP9) secretion, thereby accelerating extracellular matrix degradation in the aortic wall (91). ILF3 overexpression aggravates elastin degradation in the aorta, disrupts vascular structural integrity, and promotes AAA progression (92). ATF4 mediates M1 macrophage polarization via the SMPD3 signaling pathway, thereby amplifying local inflammatory responses (93). KLF5 exerts bidirectional regulatory effects on macrophage polarization by promoting the pro-inflammatory M1 phenotype while suppressing the anti-inflammatory M2 phenotype, thereby exacerbating AAA-related tissue injury (94). CD95L activates the caspase-8 signaling pathway, inducing inflammatory activation and extracellular matrix degradation in the aortic wall, thereby aggravating AAA pathology (95). In addition, non-coding RNAs and functional genes have also been implicated in the dynamic regulation of AAA. The olfactory receptor Olfr2, a recently identified receptor, has been shown to promote monocyte recruitment to the vascular wall by upregulating the chemokine receptor CX3CR1 on monocytes, thereby exacerbating macrophage-mediated inflammation and extracellular matrix degradation and ultimately promoting AAA development. Pharmacological inhibition of CX3CR1 using the antagonist JMS-17–2 effectively attenuates AAA progression and macrophage infiltration induced by the Olfr2 agonist octanal (96). Notably, TREM2 represents a context-dependent regulatory molecule with distinct temporal functions. In early-stage AAA, TREM2 deficiency exacerbates inflammatory injury by activating the ERK pathway, upregulating adhesion molecules and inflammatory gene expression, and increasing the proportion of Ly6C^hi pro-inflammatory monocytes. In contrast, in late-stage AAA, TREM2 deficiency disrupts the SYK–CSF1R signaling axis, promotes macrophage apoptosis, reduces extracellular matrix degradation, and ultimately attenuates disease progression, thereby exhibiting a pronounced stage-dependent bidirectional regulatory effect (97).

### 4.5 Regulatory roles of non-coding RNAs in macrophage-mediated inflammatory responses

Small non-coding RNAs have been widely demonstrated to participate in the initiation and progression of AAA. Aberrant overexpression of circCdyl promotes macrophage polarization toward the pro-inflammatory M1 phenotype, thereby exacerbating local aortic inflammation and accelerating AAA progression. Macrophage-derived circHipk3, a circular RNA, interacts with STAT3 to increase NLRP3 expression in the aorta, and also binds to SND1 to promote Ptbp1 mRNA degradation, thereby inhibiting autophagy and promoting macrophage pyroptosis, ultimately contributing to AAA pathogenesis (98).

In addition to circRNAs, microRNAs (miRNAs) have also been implicated in AAA development. miR-146a acts as a negative regulatory factor by suppressing pro-inflammatory signaling pathways involved in macrophage activation. Its deficiency promotes macrophage polarization toward the pro-inflammatory M1 phenotype, thereby aggravating local inflammatory responses in the aortic wall l (98). miR-24 targets the Chi311 gene, and by inhibiting its expression, reduces macrophage recruitment to aneurysmal lesions, decreases macrophage survival, and suppresses the production of pro-inflammatory cytokines such as IL-8 and CCL2 by monocytes and macrophages, thereby limiting inflammatory propagation at its source. In contrast, miR-33 exerts an opposing role; its deficiency leads to reduced expression of M1-associated genes in macrophages, decreased protease activity, and reduced macrophage infiltration into the aortic wall (99). miR-221-5p has been identified as a key effector molecule in M2-derived extracellular vesicles (M2-EVs) against AAA. It suppresses M1 polarization while promoting M2 polarization. Mechanistically, miR-221-5p directly binds to PARP-1 mRNA and inhibits its expression, thereby enhancing PP-1α activity and suppressing phosphorylation of the JNK/c-Jun signaling pathway. This ultimately inhibits macrophage M1 polarization, attenuates AAA formation, and exerts vascular protective effects (100).

### 4.6 Crosstalk between macrophages and other vascular cells in abdominal aortic aneurysm progression

Beyond their direct involvement in the initiation and progression of AAA, macrophages also engage in extensive crosstalk with multiple vascular cell types, which plays a pivotal regulatory role in AAA pathogenesis. PKM2 activation in T lymphocytes promotes the release of extracellular vesicles enriched in polyunsaturated phospholipids; these vesicles can be internalized by macrophages, thereby providing substrates for lipid peroxidation. In parallel, they inhibit macrophage iron efflux, leading to intracellular iron accumulation, disruption of redox homeostasis, and subsequent induction of iron-dependent lipid peroxidation. This cascade ultimately enhances macrophage migration and infiltration into the aortic wall (101). In addition, platelets regulate fibroblast behavior through paracrine signaling, thereby amplifying monocyte recruitment and activation within aneurysmal lesions. This process significantly upregulates the expression of pro-inflammatory genes in macrophages, including IL-6, IL-12B, and IL-1β, as well as genes associated with vascular remodeling, such as osteopontin, MMP9, and type I collagen. Collectively, these changes exacerbate extracellular matrix degradation and promote pathological vascular remodeling (102). These findings collectively suggest that macrophage-mediated pathological injury in AAA is not solely driven by intrinsic phenotypic dysregulation. Rather, aberrant intercellular communication between macrophages and other vascular-resident or infiltrating cells represents a critical mechanism in disease progression, and targeted modulation of these intercellular signal transduction networks may constitute a promising therapeutic strategy for future AAA intervention.

### 4.7 Progress in macrophage-targeted imaging and macrophage-directed therapy in abdominal aortic aneurysm

Given the functional alterations of macrophages in AAA, novel imaging strategies and macrophage-targeted therapeutic approaches have been progressively developed. Exitron nanoparticles represent a newly emerging contrast agent for AAA imaging. Notably, Exitron itself does not induce macrophage polarization toward either the M1 or M2 phenotype. During LPS ^+^ IFN-γ–induced M1 polarization, the uptake of Exitron by macrophages is significantly reduced; however, Exitron nanoparticles remain readily phagocytosed by infiltrating macrophages within inflammatory aneurysmal lesions *in vivo*. Following repeated administration of this contrast nanotracer, transmission electron microscopy (TEM) reveals substantial accumulation of Exitron nanoparticles within the adventitial regions of the aneurysmal aortic wall, particularly in areas with the highest inflammatory activity, where they are readily phagocytosed by infiltrating macrophages. Accordingly, computed tomography (CT) signal intensity can indirectly reflect macrophage content within the aneurysmal wall, and Exitron-derived signal intensity has been shown to predict subsequent aneurysm expansion (100).

In terms of therapeutic strategies, biomimetic nanoparticles based on macrophage membranes have attracted considerable attention due to their enhanced targeting capability. A multifunctional nanotherapeutic platform, MPB-RLZ@MM nanoparticles, has been developed to exploit the intrinsic targeting properties of macrophage membranes. This system effectively suppresses M1 macrophage polarization while promoting M2 polarization. Mechanistically, MPB-RLZ@MM nanoparticles first activate the Nrf2-mediated antioxidant pathway, thereby inducing the expression of antioxidant genes such as Cat, Sod1, Nqo1, Tgfb1, and Il10 to eliminate excessive ROS. Subsequently, they inhibit the NF-κB inflammatory signal transduction pathway, downregulating pro-inflammatory genes including Tnf, Il1b, and Nos2, thereby reducing the release of pro-inflammatory mediators (103).

Most of the aforementioned studies primarily focus on circulating macrophages. With the advancement of research, a recently identified subset of vascular tissue-resident macrophages (VRMs) located in the adventitial layer, characterized by the expression of LYVE1, has gained increasing attention. This Lyve1^+^ VRM population exerts protective effects against AAA through the secretion of the extracellular matrix protein SPARCL1 (Sc1). SPARCL1 inhibits aberrant lymphangiogenesis and tertiary lymphoid structure (TLS) formation within the aortic wall by binding to and blocking FGF2, thereby attenuating aneurysm progression (10). Conversely, deficiency of Sc1 in Lyve1^+^ VRMs promotes pathological lymphangiogenesis, upregulates CXCL13 and LTβR expression, induces TLS formation, and consequently exacerbates AAA development (10). However, the role of MacAIR in AAA remains unclear, and no related studies have yet been reported.

## 5 The role of macrophages in the pathogenesis of aortic dissection

AD is one of the most critical and life-threatening forms of aortic disease, characterized by an intimal tear that allows blood to enter the medial layer of the aortic wall, forming a false lumen that compresses the true lumen and disrupts blood flow. The disease exhibits abrupt onset, rapid progression, and extremely high mortality. Its pathogenesis is associated with increased mechanical stress on the aortic wall or structural damage of the medial layer (104), with common risk factors including hypertension (105) atherosclerosis, and Marfan syndrome (106). Notably, extensive infiltration of monocytes/macrophages is consistently observed across all etiologies of AD (107), and the number of macrophages far exceeds that of other immune cell populations (97, 108–110). β-aminopropionitrile (BAPN), a lysyl oxidase inhibitor that weakens the aortic wall by impairing collagen and elastin crosslinking, is commonly used to establish murine models of aortic dissection. In a β-aminopropionitrile (BAPN)-induced murine model, in which BAPN inhibits lysyl oxidase–mediated collagen and elastin crosslinking, three macrophage subsets—pro-inflammatory, reparative, and resident macrophages—have been identified (109). Infiltrating macrophages are primarily derived from circulating monocytes and contribute to disease progression by secreting matrix metalloproteinases, pro-inflammatory cytokines, and chemokines, thereby exacerbating vascular smooth muscle cell apoptosis, promoting medial degeneration, and ultimately leading to aortic delamination or rupture (108).

Macrophages exhibit dual roles in both inflammation and tissue repair. Their inflammatory infiltration contributes to extracellular matrix degradation, loss or phenotypic switching of VSMCs, and ultimately the initiation of dissection (107). Upon microenvironmental stimulation, quiescent M0 macrophages differentiate into classically activated pro-inflammatory M1 macrophages or alternatively activated anti-inflammatory M2 macrophages (111). M1 macrophages drive pathological vascular remodeling by secreting matrix metalloproteinases (MMPs), TNF-α, and interleukins such as IL-1, IL-6, and IL-12, thereby promoting elastin fragmentation and medial destruction (112). In contrast, M2 macrophages suppress inflammation and facilitate tissue repair and healing within dissected aortic regions (113). Elevated ceramide levels promote M1 polarization and increase the release of MMP2 and MMP9, thereby accelerating dissection rupture (114). Prostaglandin E2 (PGE2) and leukotrienes (LTB4, LTD4, LTE4) also activate macrophages and promote M1 polarization (115), whereas CD31+ signals can induce M1-to-M2 transition and support aortic repair (120). Bruton’s tyrosine kinase (BTK) participates in macrophage polarization regulation and limits AD progression (116), while liraglutide suppresses M1 polarization by inhibiting CXCL3 expression via the PI3K/AKT signal transduction pathway (117).

Under hypoxic conditions, activation of HIF-1α promotes M1 polarization and aggravates disease severity. Concurrently, downstream target ADAM17 is upregulated, along with increased secretion of MMP2 and MMP9, accelerating degradation of extracellular matrix components in the aortic wall (118). Thus, the HIF-1α–ADAM17 axis represents a key pathway in macrophage-mediated AD progression (119). In addition, the TREM2/TYROBP signal transduction pathway plays an inhibitory role in macrophage activation, and TREM2 activation has been shown to protect against AD progression (103). In the Ang II–AT1R–YAP axis, YAP overexpression promotes M2 polarization, whereas YAP phosphorylation shifts macrophages toward the pro-inflammatory CD68^+^CD86^+^ M1 phenotype, increasing secretion of IL-6, endothelin-1 (ET-1), and MMP9, thereby accelerating intimal destruction and false lumen formation. Intervention with angiotensin receptor blockers (ARBs) increases M2 macrophage infiltration and restores an anti-inflammatory phenotype (120). In macrophages lacking HIF-1α, expression and activity of MMP2 and MMP9 are significantly reduced (119).

Metabolic reprogramming is also critically involved in macrophage-driven AD. Elevated succinate levels are observed in M1 macrophages from AD patients, and the p38α–CREB–OGDH axis regulates succinate homeostasis to delay disease progression (121). In parallel, RBM15 is highly expressed under glycolytic conditions and is associated with enhanced M1 polarization (122). Macrophage-derived legumain (LGMN) binds to integrin αvβ3 on vascular smooth muscle cells, inhibiting Rho GTPase activation, downregulating VSMC differentiation markers, and ultimately promoting vascular degeneration and thoracic AD progression (123). Increased expression of ACKR1 in the vascular wall activates the ACKR1/NF-κB/SPP1 axis, facilitating macrophage recruitment (124). Macrophages in diseased regions exhibit elevated ADAM17 expression, which promotes TNF-α maturation and secretion, amplifying inflammatory responses and immune cell infiltration. Through the EGFR–MEK–ERK pathway, ADAM17 further enhances MMP2/MMP9 expression and activity, accelerating extracellular matrix degradation and elastin fragmentation (119). Moreover, the dsDNA–STING–IRF3 axis induces inflammatory gene expression in VSMCs, including CCL2, IL-6, and IFN-β, thereby contributing to immune cell recruitment and amplification of vascular inflammation (125, 126).

Angiotensin II–induced upregulation of calpain-2 activates the Calpain2–Talin1–ITGAV axis, disrupting endothelial integrity; macrophage-specific inhibition of Capns1 confers vascular protection (127). In the STAT3 pathway, macrophage-specific deletion of SOCS3 enhances STAT3 activation, promotes M1 polarization, and exacerbates vascular injury (128). Notch–RBPJ signaling in macrophages drives circulating monocyte-derived M1 polarization, while RBPJ-mediated upregulation of PDK1 accelerates glycolysis and vascular pathology (129). RUNX1 overexpression in adipose-associated contexts promotes M1 polarization via NF-κB signal transduction (130). NF-κB signal transduction is a central pathway in AD pathogenesis, leading to p65 phosphorylation and tissue injury (118). Upstream mediators such as 12-HETE/BLT2 promote IL-6 release and disease initiation (131). Additionally, SNO-Septin2 regulates extracellular matrix degradation via the TIAM1–RAC1 axis (132), while p38 MAPK–HSP27 (133) and miR-92a-3p/FOSL2/NF-κB (134) pathways further drive pro-inflammatory macrophage polarization.

Collectively, these findings highlight that signal transduction pathways are central regulators of AD pathogenesis and elucidate key molecular mechanisms underlying disease progression. However, most identified pathways have been studied primarily in circulating monocyte-derived macrophages, whereas the role of aortic RTMs remains poorly understood. Current evidence is limited, with only isolated reports such as JAK2V617F mutation–driven pathogenic activation of adventitial resident macrophages, which can initiate early maladaptive vascular remodeling and later amplify inflammation by recruiting circulating monocytes (108, 135). Therefore, greater attention should be directed toward the pathogenic roles and dynamics of RTMs in AD.

Beyond direct macrophage effects, cell–cell interaction analyses have revealed that macrophages establish active communication networks with T cells through enhanced co-stimulatory signal transduction, jointly driving inflammatory responses and cytotoxic activity and thereby exacerbating vascular injury (136). The inflamed vascular microenvironment is characterized by extensive immune cell infiltration and intercellular crosstalk, indicating a conceptual shift in AD pathogenesis from a purely “mechanical dissection” model to an “active immune-mediated destruction” paradigm. With continued advances in research, additional functionally relevant macrophage subsets have been identified. Recent studies have shown that BASP1^+^ macrophages, derived from infiltrating BASP1^+^ monocytes, represent a key population driving early vascular wall destruction and initiating inflammatory cascades in AD (137). Furthermore, multi-omics analyses have identified a lipid metabolism–dysregulated FABP5^+^ macrophage subset in thoracic aortic dissection (TAAD), which links metabolic imbalance to tissue destruction by promoting foam cell formation, activating the NLRP3 inflammasome, inducing pyroptosis, and secreting potent pro-inflammatory mediators, thereby driving inflammatory storm, medial degeneration, and pathological fibrosis leading to vascular rupture (108). However, it should be noted that the mean age of patients in this study was approximately 53years, and whether these findings are generalizable to all TAAD populations remains uncertain.

At present, most studies remain focused on circulating monocyte-derived macrophages, whereas knowledge regarding aortic RTMs is still limited. Importantly, resident macrophages may not be exclusively protective; under specific genetic mutations, aging, or chronic inflammatory conditions, they may also acquire pathogenic inflammatory phenotypes and actively participate in early vascular remodeling and monocyte recruitment. Future investigations into AD should therefore prioritize elucidating the spatiotemporal heterogeneity and dynamic lineage plasticity of macrophage populations derived from distinct origins.

## 6 The role of macrophages in vascular aging

Vascular aging is increasingly recognized as a fundamental pathological basis underlying the initiation and progression of aortic diseases. Its major hallmarks include endothelial dysfunction, VSMC senescence, ECM remodeling, and persistent chronic low-grade inflammation (138). Recent studies have identified immune aging, particularly macrophage dysfunction, as a critical mechanistic link connecting aging with aortic pathology (139). With advancing age, the organism gradually develops a state termed “inflammaging,” characterized by sustained low-level systemic inflammation and persistent pro-inflammatory activation of macrophages (140). Oxidative stress, mitochondrial dysfunction, and metabolic disturbances collectively promote chronic activation of the NLRP3 inflammasome, leading to excessive maturation and release of IL-1β and IL-18, which further amplify inflammatory responses and are associated with increased expression of TNF-α and IL-6, thereby exacerbating chronic inflammation within the aortic wall, accelerating elastic fiber fragmentation, and enhancing extracellular matrix degradation (141). Tyrrell et al. (142) further proposed that inflammaging not only contributes to chronic vascular injury but also reshapes the interactions between immune cells and vascular cells, thereby driving the progression of degenerative vascular disorders, including aortic aneurysm, aortic dissection, and atherosclerosis.

In addition to persistent inflammatory activation, aged macrophages undergo profound immunometabolic reprogramming (143). Accumulating evidence suggests that senescent macrophages preferentially rely on glycolytic metabolism and are accompanied by reduced NAD^+^ levels, increased CD38 expression, and excessive accumulation of mitochondrial reactive oxygen species (ROS), all of which contribute to the maintenance of M1-like pro-inflammatory polarization (144). Yarbro et al. (145) described aged macrophages as exhibiting a dual phenotype characterized by “elevated basal inflammatory activity coupled with impaired inflammatory resolution,” thereby maintaining the vasculature in a chronic low-grade inflammatory state. Sustained activation of the NLRP3 inflammasome may also trigger pyroptosis, further amplifying local inflammatory responses and accelerating vascular structural degeneration (140, 141). Consequently, macrophage-associated inflammatory metabolic dysfunction is now considered a major driving force in vascular aging.

Significant alterations in macrophage abundance and phenotypic composition have also been observed in aged aortic tissues. In aged murine aortas, macrophage accumulation is markedly increased, accompanied by upregulation of M1-associated markers, including CD38, CD86, and CD80, whereas M2-associated markers such as EGR2, CD206, and CD163 are significantly reduced. Notably, Nox4 deficiency partially reverses aging-associated macrophage polarization imbalance (139). Concurrently, aging vessels exhibit increased expression of immune evasion molecules, including Cd47, Sirpa, Cd24a, and Clec4a, which impair macrophage-mediated clearance of senescent cells. Vascular aging is further characterized by profound imbalance among macrophage subsets, with a decline in tissue-resident Lyve1^+^ macrophages and a marked increase in infiltrating bone marrow–derived Lyve1^-^/Ccr2^+^ inflammatory macrophages. Bone marrow–derived macrophages preferentially express pro-inflammatory genes such as Fgr and Myo1f, whereas RTMs exhibit higher expression of fibrosis-associated genes including Cd209, Timp2, and Ccl8. Collectively, these alterations promote the transition of adventitial macrophages toward a pro-inflammatory phenotype, thereby contributing to vascular inflammaging and fibrosis (146). The observation that Nox4 deficiency partially restores this polarization imbalance further highlights the central role of oxidative stress in vascular immune aging, as Nox4 contributes to the generation of reactive oxygen species (ROS), particularly hydrogen peroxide (H_2_O_2_) (147). Recent advances in single-cell transcriptomic technologies have further revealed the dynamic remodeling of macrophage lineage architecture during vascular aging (7). Under physiological conditions, Lyve1^+^ RTMs predominate within the aortic adventitia and are primarily responsible for maintaining immune homeostasis, facilitating tissue repair, and suppressing excessive inflammation.9 However, aging is associated with a substantial reduction in this resident macrophage population, accompanied by extensive infiltration of bone marrow–derived Lyve1^-^/Ccr2^+^ inflammatory macrophages (7). These recruited macrophages highly express pro-inflammatory genes such as Fgr and Myo1f, thereby amplifying inflammatory chemotaxis and immune activation, whereas resident macrophages preferentially express genes associated with fibrotic remodeling and tissue repair, including Cd209, Timp2, and Ccl8.7 As the balance between these macrophage populations becomes disrupted, the aortic adventitia gradually shifts from a homeostatic repair-oriented immune microenvironment toward a persistently pro-inflammatory state, ultimately promoting vascular fibrosis, extracellular matrix remodeling, and loss of biomechanical stability of the aortic wall, thereby increasing susceptibility to aortic aneurysm and aortic dissection (7, 142).

Collectively, growing evidence indicates that macrophages are not merely passive participants in vascular aging but active drivers of aortic disease progression (142, 148). Accordingly, therapeutic strategies targeting macrophage immunometabolism, NLRP3 inflammasome activation, and senescent cell clearance (senolysis) are emerging as promising approaches for delaying vascular aging and preventing aortic diseases (140, 144). For instance, inhibition of NLRP3 inflammasome activation, restoration of NAD^+^ metabolic homeostasis, and enhancement of macrophage-mediated clearance of senescent cells have all been proposed as potential strategies to alleviate chronic inflammatory conditions within the aortic wall and attenuate age-related vascular degeneration (141, 145).

## 7 The role of macrophages in vascular inflammation

Macrophages are central immune effectors in vascular inflammation and the progression of aortic diseases. Traditionally, macrophages within vascular lesions were considered to arise predominantly from recruited circulating monocytes. However, recent advances in single-cell RNA sequencing, lineage tracing, and spatial transcriptomics have revealed substantial heterogeneity among aortic macrophage populations, including monocyte-derived macrophages, VRMs located in the adventitia, and macrophages associated with the Mac^AIR^. These distinct macrophage subsets exert divergent, and at times opposing, functions in tissue homeostasis, inflammatory amplification, and vascular repair processes (149, 150). Monocyte-derived macrophages represent the predominant inflammatory macrophage population during vascular injury. Under conditions such as hyperlipidemia, disturbed shear stress, vascular injury, or angiotensin II (Ang II) stimulation, circulating CCR2^+^Ly6C^high^ monocytes are recruited to the aortic wall through chemokine pathways including CCL2/CCR2 and subsequently differentiate into inflammatory macrophages156. These cells characteristically express high levels of IL-1β, TNF-α, MMP2 and MMP9, and ROS-related mediators, thereby promoting elastic fiber fragmentation, extracellular matrix degradation, and destabilization of the vascular wall, ultimately contributing to aneurysm formation and aortic dissection (151). In atherosclerosis, monocyte-derived macrophages also internalize ox-LDL and differentiate into foam cells while sustaining the chronic inflammatory milieu through persistent cytokine secretion. Emerging evidence further suggests that these macrophages acquire features of “trained immunity,” whereby prior inflammatory stimulation induces a long-lasting pro-inflammatory phenotype, potentially contributing to age-associated chronic vascular inflammation (148).

In addition to recruited macrophages, the aortic adventitia harbors a substantial population of RTMs. Lineage-tracing studies by Weinberger and colleagues demonstrated that a subset of resident aortic macrophages originates from embryonic EMPs) derived from the yolk sac and is maintained locally through self-renewal rather than continuous bone marrow replenishment (149). These resident macrophages typically express Lyve1, Stab1, and Mrc1 (CD206), and are primarily involved in anti-inflammatory regulation, clearance of apoptotic cells, and maintenance of vascular homeostasis (149, 152). Under physiological conditions, they preserve aortic integrity by limiting inflammatory spread, regulating collagen turnover, and removing necrotic debris. However, persistent inflammatory stimulation can drive functional reprogramming of resident macrophages toward pro-inflammatory phenotypes, thereby contributing to adventitial remodeling and vascular fibrosis in concert with recruited macrophages (153). Compared with monocyte-derived macrophages, Mac^AIR^ populations appear to participate earlier in lesion initiation and may function as local inflammatory sentinels during the early stages of disease development (6, 7).

Macrophage populations of different origins do not function independently but instead form highly interconnected regulatory networks through cytokines, chemokines, and metabolic signaling pathways. Monocyte-derived macrophages are generally associated with inflammatory amplification and tissue destruction, whereas resident macrophages preferentially participate in inflammation resolution and tissue repair; nevertheless, both populations undergo substantial functional remodeling under chronic inflammatory conditions (150, 152). Consequently, precise immunomodulatory strategies targeting specific macrophage subsets may represent a promising therapeutic direction for vascular inflammatory diseases.

## 8 Conclusion

Macrophages play central roles in aortic physiology and pathophysiology. They participate in immune surveillance, inflammatory regulation, extracellular matrix remodeling, vascular homeostasis, and tissue repair. Recent studies have shown that different macrophage populations, including circulating monocyte-derived macrophag RTMs, and Mac^AIR,^ actively shape the aortic immune microenvironment. These macrophage subsets also interact closely with vascular and immune cells during disease progression.

Macrophages contribute to the development of atherosclerosis, abdominal aortic aneurysm, and aortic dissection through multiple mechanisms. These mechanisms include inflammatory amplification, foam cell formation, programmed cell death, extracellular matrix degradation, and metabolic reprogramming of immune cells. Several signaling pathways regulate these processes. These pathways include NF-κB, JAK/STAT, PI3K/AKT/mTOR, cGAS–STING, NLRP3 inflammasome, and HIF-1α. At the same time, macrophage-targeted therapies have shown promising potential. These strategies include nanomedicine-based delivery systems, RNA therapies, metabolic reprogramming approaches, and macrophage-targeted imaging technologies.

Recent advances in single-cell sequencing, spatial transcriptomics, lineage tracing, and multi-omics technologies have greatly improved our understanding of macrophage heterogeneity and spatiotemporal dynamics in aortic diseases. However, most current studies still focus mainly on circulating monocyte-derived macrophages. The physiological and pathological roles of RTMs and Mac^AIR^ remain insufficiently understood. In addition, most available evidence comes from animal models. Therefore, future studies should further integrate single-cell and spatial omics findings with the clinical characteristics of human aortic diseases. Future directions will likely provide a more comprehensive understanding of the spatiotemporal evolution and functional interactions of macrophage subsets in aortic diseases. A deeper understanding of macrophage heterogeneity and immunometabolic regulation may help clarify the immunopathological mechanisms underlying aortic disorders. These findings may also support the development of novel biomarkers and precision macrophage-targeted therapeutic strategies for aortic diseases.

## References

[1] Libby P . The changing landscape of atherosclerosis. Nature. (2021) 592:524–33. doi: 10.1038/s41586-021-03392-8 33883728

[2] Williams JW Giannarelli C Rahman A Randolph GJ Kovacic JC . Macrophage biology, classification and phenotype in cardiovascular disease: JACC macrophage series (part I). J Am Coll Cardiol. (2018) 72:2166–80. doi: 10.1016/j.jacc.2018.08.2148 30360826 PMC6209330

[3] Roy P Orecchioni M Ley K . How the immune system shapes atherosclerosis: Roles of innate and adaptive immunity. Nat Rev Immunol. (2022) 22:251–65. doi: 10.1038/s41577-021-00584-1 34389841 PMC10111155

[4] Quintana RA Taylor WR . Cellular mechanisms of aortic aneurysm formation. Circ Res. (2019) 124:607–18. doi: 10.1161/CIRCRESAHA.118.313187 30763207 PMC6383789

[5] Guilliams M Mildner A Yona S . Developmental and functional heterogeneity of monocytes. Immunity. (2018) 49:595–613. doi: 10.1016/j.immuni.2018.10.005 30332628

[6] Cochain C Vafadarnejad E Arampatzi P Pelisek J Winkels H Ley K . Single-cell RNA-seq reveals the transcriptional landscape and heterogeneity of aortic macrophages in murine atherosclerosis. Circ Res. (2018) 122:1661–74. doi: 10.1161/CIRCRESAHA.117.312509 29545365

[7] Winkels H Ehinger E Vassallo M Buscher K Dinh H Kobiyama K . Atlas of the immune cell repertoire in mouse atherosclerosis defined by single-cell RNA-sequencing and mass cytometry. Circ Res. (2018) 122:1675–88. doi: 10.1161/CIRCRESAHA.117.312513 29545366 PMC5993603

[8] Kim K Shim D Lee JS Zaitsev K Williams JW Kim K-W . Transcriptome analysis reveals non-foamy rather than foamy plaque macrophages are pro-inflammatory in atherosclerotic murine models. Circ Res. (2018) 123:1127–42. doi: 10.1161/CIRCRESAHA.118.312804 30359200 PMC6945121

[9] Two Distinct Interstitial Macrophage Populations Coexist Across Tissues in Specific Subtissular Niches | Science. Available online at: https://www.science.org/doi/10.1126/science.aau0964 (Accessed May 23, 2026). 10.1126/science.aau0964 30872492

[10] Chen M-H Hua Y-J Li Y Lei Y Zhou S-Y Hu X-D . Sparcl1 mitigates abdominal aortic aneurysm through inhibiting lymphangiogenesis-mediated TLS formation. Nat Immunol. (2026) 27:738–49. doi: 10.1038/s41590-026-02454-1 41760906

[11] Williams JW Zaitsev K Kim K-W Ivanov S Saunders BT Schrank PR . Limited proliferation capacity of aorta intima resident macrophages requires monocyte recruitment for atherosclerotic plaque progression. Nat Immunol. (2020) 21:1194–204. doi: 10.1038/s41590-020-0768-4 32895539 PMC7502558

[12] Swirski FK Nahrendorf M . Cardioimmunology: The immune system in cardiac homeostasis and disease. Nat Rev Immunol. (2018) 18:733–44. doi: 10.1038/s41577-018-0065-8 30228378

[13] Murray PJ Allen JE Biswas SK Fisher EA Gilroy DW Goerdt S . Macrophage activation and polarization: Nomenclature and experimental guidelines. Immunity. (2014) 41:14–20. doi: 10.1016/j.immuni.2014.06.008 25035950 PMC4123412

[14] Mosser DM Hamidzadeh K Goncalves R . Macrophages and the maintenance of homeostasis. Cell Mol Immunol. (2021) 18:579–87. doi: 10.1038/s41423-020-00541-3 32934339 PMC7491045

[15] Mass E Nimmerjahn F Kierdorf K Schlitzer A . Tissue-specific macrophages: How they develop and choreograph tissue biology. Nat Rev Immunol. (2023) 23:563–79. doi: 10.1038/s41577-023-00848-y 36922638 PMC10017071

[16] Hernandez GE Ma F Martinez G Firozabadi NB Salvador J Juang LJ . Aortic intimal resident macrophages are essential for maintenance of the non-thrombogenic intravascular state. Nat Cardiovasc Res. (2022) 1:67–84. doi: 10.1038/s44161-021-00006-4 35599984 PMC9121812

[17] Zhang L . Contribution of resident and recruited macrophages in vascular physiology and pathology. Curr Opin Hematol. (2018) 25:196–203. doi: 10.1097/MOH.0000000000000421 29438258

[18] Chaintreuil P Kerreneur E Bourgoin M Savy C Favreau C Robert G . The generation, activation, and polarization of monocyte-derived macrophages in human Malignancies. Front Immunol. (2023) 14:1178337. doi: 10.3389/fimmu.2023.1178337 37143666 PMC10151765

[19] Li Q Wang M Zhang S Jin M Chen R Luo Y . Single-cell RNA sequencing in atherosclerosis: Mechanism and precision medicine. Front Pharmacol. (2022) 13:977490. doi: 10.3389/fphar.2022.977490 36267275 PMC9576927

[20] Park MD Silvin A Ginhoux F Merad M . Macrophages in health and disease. Cell. (2022) 185:4259–79. doi: 10.1016/j.cell.2022.10.007 36368305 PMC9908006

[21] Psaltis PJ Puranik AS Spoon DB Chue CD Hoffman SJ Witt TA . Characterization of a resident population of adventitial macrophage progenitor cells in postnatal vasculature. Circ Res. (2014) 115(3):364–75. doi: 10.1161/CIRCRESAHA.115.303299 24906644

[22] de Winther MPJ Bäck M Evans P Gomez D Goncalves I Jørgensen HF . Translational opportunities of single-cell biology in atherosclerosis. Eur Heart J. (2022) 44:1216–30. doi: 10.1093/eurheartj/ehac686 36478058 PMC10120164

[23] Park I Goddard ME Cole JE Zanin N Lyytikäinen L-P Lehtimäki T . C-type lectin receptor CLEC4A2 promotes tissue adaptation of macrophages and protects against atherosclerosis. Nat Commun. (2022) 13:215. doi: 10.1038/s41467-021-27862-9 35017526 PMC8752790

[24] Sebastian A Hum NR McCool JL Wilson SP Murugesh DK Martin KA . Single-cell RNA-seq reveals changes in immune landscape in post-traumatic osteoarthritis. Front Immunol. (2022) 13. doi: 10.3389/fimmu.2022.938075 35967299 PMC9373730

[25] Williamson AE Liyanage S Hassanshahi M Dona MSI Toledo-Flores D Tran DXA . Discovery of an embryonically derived bipotent population of endothelial-macrophage progenitor cells in postnatal aorta. Nat Commun. (2024) 15:7097. doi: 10.1038/s41467-024-51637-7 39154007 PMC11330468

[26] Ensan S Li A Besla R Degousee N Cosme J Roufaiel M . Self-renewing resident arterial macrophages arise from embryonic CX3CR1+ precursors and circulating monocytes immediately after birth. Nat Immunol. (2016) 17:159–68. doi: 10.1038/ni.3343 26642357

[27] Ginhoux F Guilliams M . Tissue-resident macrophage ontogeny and homeostasis. Immunity. (2016) 44:439–49. doi: 10.1016/j.immuni.2016.02.024 26982352

[28] Samokhvalov IM Samokhvalova NI Nishikawa S . Cell tracing shows the contribution of the yolk sac to adult haematopoiesis. Nature. (2007) 446:1056–61. doi: 10.1038/nature05725 17377529

[29] Mass E Ballesteros I Farlik M Halbritter F Günther P Crozet L . Specification of tissue-resident macrophages during organogenesis. Science. (2016) 353:aaf4238. doi: 10.1126/science.aaf4238 27492475 PMC5066309

[30] Hashimoto D Chow A Noizat C Teo P Beasley MB Leboeuf M . Tissue-resident macrophages self-maintain locally throughout adult life with minimal contribution from circulating monocytes. Immunity. (2013) 38:792–804. doi: 10.1016/j.immuni.2013.04.004 23601688 PMC3853406

[31] Bain CC Bravo-Blas A Scott CL Perdiguero EG Geissmann F Henri S . Constant replenishment from circulating monocytes maintains the macrophage pool in the intestine of adult mice. Nat Immunol. (2014) 15:929–37. doi: 10.1038/ni.2967 25151491 PMC4169290

[32] Lim HY Lim SY Tan CK Thiam CH Goh CC Carbajo D . Hyaluronan receptor LYVE-1-expressing macrophages maintain arterial tone through hyaluronan-mediated regulation of smooth muscle cell collagen. Immunity. (2018) 49:326–341.e7. doi: 10.1016/j.immuni.2018.06.008 30054204

[33] Weijts B Demmers JAA Robin C . Blood vessel-resident macrophages safeguard blood and vessel integrity in zebrafish. Nat Immunol. (2026) 27(5):975–84. doi: 10.1038/s41590-026-02481-y 41917203 PMC13132727

[34] Jones PW Mallat Z Nus M . T-cell/B-cell interactions in atherosclerosis. Arterioscler Thromb Vasc Biol. (2024) , 44(7):1502–11. doi: 10.1161/ATVBAHA.124.319845 38813700 PMC11208060

[35] Mantovani A Garlanda C Locati M . Macrophage diversity and polarization in atherosclerosis. Arteriosclerosis Thrombosis Vasc Biol. (2009) 29(10):1419–23. doi: 10.1161/ATVBAHA.108.180497 19696407

[36] Libby P Ridker PM Hansson GK . Progress and challenges in translating the biology of atherosclerosis. Nature. (2011) 473:317–25. doi: 10.1038/nature10146 21593864

[37] Moore KJ Sheedy FJ Fisher EA . Macrophages in atherosclerosis: A dynamic balance. Nat Rev Immunol. (2013) 13:709–21. doi: 10.1038/nri3520 23995626 PMC4357520

[38] Swirski FK Libby P Aikawa E Alcaide P Luscinskas FW Weissleder R . Ly-6C^hi^ monocytes dominate hypercholesterolemia-associated monocytosis and give rise to macrophages in atheromata. J Clin Invest. (2007) 117:195–205. doi: 10.1172/JCI29950 17200719 PMC1716211

[39] Hilgendorf I Swirski FK Robbins CS . Monocyte fate in atherosclerosis. Arterioscler Thromb Vasc Biol. (2015) 35(2):272–9. doi: 10.1161/ATVBAHA.114.303565 25538208

[40] Tacke F Alvarez D Kaplan TJ Jakubzick C Spanbroek R Llodra J . Monocyte subsets differentially employ CCR2, CCR5, and CX3CR1 to accumulate within atherosclerotic plaques. J Clin Invest. (2007) 117:185–94. doi: 10.1172/JCI28549 17200718 PMC1716202

[41] Wu S Zhao S Hai L Yang Z Wang S Cui D . Macrophage polarization regulates the pathogenesis and progression of autoimmune diseases. Autoimmun Rev. (2025) 24:103820. doi: 10.1016/j.autrev.2025.103820 40268127

[42] Ji Y Li X Yao X Sun J Yi J Shen Y . Macrophage polarization: Molecular mechanisms, disease implications, and targeted therapeutic strategies. Front Immunol. (2025) 16. doi: 10.3389/fimmu.2025.1732718 41459510 PMC12740920

[43] Murray PJ Wynn TA . Protective and pathogenic functions of macrophage subsets. Nat Rev Immunol. (2011) 11:723–37. doi: 10.1038/nri3073 21997792 PMC3422549

[44] Zhang L Li J Kou Y Shen L Wang H Wang Y . Mechanisms and treatment of atherosclerosis: Focus on macrophages. Front Immunol. (2024) 15. doi: 10.3389/fimmu.2024.1490387 39569201 PMC11576186

[45] Libby P . Inflammation during the life cycle of the atherosclerotic plaque. 117(13):2525–36. doi: 10.1093/cvr/cvab303 PMC8783385 34550337

[46] Libby P . Inflammation and the pathogenesis of atherosclerosis. VascPharmacol. (2024) 154:107255. doi: 10.1016/j.vph.2023.107255 38157682

[47] Varga T Mounier R Horvath A Cuvellier S Dumont F Poliska S . Highly dynamic transcriptional signature of distinct macrophage subsets during sterile inflammation, resolution, and tissue repair. 196(11):4771–82. doi: 10.4049/jimmunol.1502490 27183604

[48] Chen S Yang J Wei Y Wei X . Epigenetic regulation of macrophages: From homeostasis maintenance to host defense. Cell Mol Immunol. (2020) 17:36–49. doi: 10.1038/s41423-019-0315-0 31664225 PMC6952359

[49] Feng X Chen W Ni X Little PJ Xu S Tang L . Metformin, macrophage dysfunction and atherosclerosis. Front Immunol. (2021) 12:682853. doi: 10.3389/fimmu.2021.682853 34163481 PMC8215340

[50] Arabpour M Saghazadeh A Rezaei N . Anti-inflammatory and M2 macrophage polarization-promoting effect of mesenchymal stem cell-derived exosomes. Int Immunopharmacol. (2021) 97:107823. doi: 10.1016/j.intimp.2021.107823 34102486

[51] Kadl A Meher AK Sharma PR Lee MY Doran AC Johnstone SR . Identification of a novel macrophage phenotype that develops in response to atherogenic phospholipids via Nrf2. Circ Res. (2010) 107:737–46. doi: 10.1161/CIRCRESAHA.109.215715 20651288 PMC2941538

[52] Domschke G Gleissner CA . CXCL4-induced macrophages in human atherosclerosis. Cytokine. (2019) 122:154141. doi: 10.1016/j.cyto.2017.08.021 28899579

[53] Poznyak AV Nikiforov NG Starodubova AV Popkova TV Orekhov AN . Macrophages and foam cells: Brief overview of their role, linkage, and targeting potential in atherosclerosis. Biomedicines. (2021) 9:1221. doi: 10.3390/biomedicines9091221 34572406 PMC8468383

[54] Eshghjoo S Kim DM Jayaraman A Sun Y Alaniz RC . Macrophage polarization in atherosclerosis. Genes. (2022) 13, 756.55:2703. doi: 10.3390/genes13050756 PMC9142092 35627141

[55] Linton MF Moslehi JJ Babaev VR . Akt signaling in macrophage polarization, survival, and atherosclerosis. Int J Mol Sci. (2019) 20. doi: 10.3390/ijms20112703 31159424 PMC6600269

[56] Zhang X Evans TD Chen S Sergin I Stitham J Jeong SJ . Loss of macrophage mTORC2 drives atherosclerosis via FoxO1 and IL-1β signaling. 133(3):200–19. doi: 10.1161/CIRCRESAHA.122.321542 PMC10527041 37350264

[57] Rui H Yu H Chi K Han Z Zhu W Zhang J . ALDH2 deficiency augments atherosclerosis through the USP14-cGAS-dependent polarization of proinflammatory macrophages. Redox Biol. (2024) 76:103318. doi: 10.1016/j.redox.2024.103318 39178733 PMC11388276

[58] Zheng Y Li Y Ran X Wang D Zheng X Zhang M . Mettl14 mediates the inflammatory response of macrophages in atherosclerosis through the NF-κB/IL-6 signaling pathway. Cell Mol Life Sci CMLS. (2022) 79:311. doi: 10.1007/s00018-022-04331-0 35598196 PMC9124663

[59] Macrophage-derived FGFR1 drives atherosclerosis through PLCγ-mediated activation of NF-κB inflammatory signalling pathway | cardiovascular research | oxford academic. Available online at: https://academic.oup.com/cardiovascres/article/120/12/1385/7688867?login=false (Accessed May 11, 2026). 10.1093/cvr/cvae131 38842387

[60] Li Z Li X Shen R Wang Y Yu J Pan C . Interleukin-38 ameliorates atherosclerosis by inhibiting macrophage M1-like polarization and apoptosis. Biomolecules. (2025) 15:1741. doi: 10.3390/biom15121741 41463394 PMC12731223

[61] Hou P Fang J Liu Z Shi Y Agostini M Bernassola F . Macrophage polarization and metabolism in atherosclerosis. Cell Death Dis. (2023) 14:691. doi: 10.1038/s41419-023-06206-z 37863894 PMC10589261

[62] Grootaert MOJ Bennett MR . Vascular smooth muscle cells in atherosclerosis: Time for a re-assessment. Cardiovasc Res. (2021) 117:2326–39. doi: 10.1093/cvr/cvab046 33576407 PMC8479803

[63] Ma J Zhang H Chen Y Liu X Tian J Shen W . The role of macrophage iron overload and ferroptosis in atherosclerosis. Biomolecules. (2022) 12:1702. doi: 10.3390/biom12111702 36421722 PMC9688033

[64] Lee-Rueckert M Jauhiainen M Kovanen PT Escolà-Gil JC . Lipids and lipoproteins in the interstitial tissue fluid regulate the formation of dysfunctional tissue-resident macrophages: Implications for atherogenic, tumorigenic, and obesogenic processes. Semin Cancer Biol. (2025) 114:104–27. doi: 10.1016/j.semcancer.2025.06.008 40545184

[65] Wei Y Lan B Zheng T Yang L Zhang X Cheng L . GSDME-mediated pyroptosis promotes the progression and associated inflammation of atherosclerosis. Nat Commun. (2023) 14:929. doi: 10.1038/s41467-023-36614-w 36807553 PMC9938904

[66] Fan X Han J Zhong L Zheng W Shao R Zhang Y . Macrophage-derived GSDMD plays an essential role in atherosclerosis and cross talk between macrophages via the mitochondria-STING-IRF3/NF-κB axis. Arterioscler Thromb Vasc Biol. (2024) 44:1365–78. doi: 10.1161/ATVBAHA.123.320612 38695170

[67] Lv J-J Wang H Zhang C Zhang T-J Wei H-L Liu Z-K . CD147 sparks atherosclerosis by driving M1 phenotype and impairing efferocytosis. Circ Res. (2024) 134:165–85. doi: 10.1161/CIRCRESAHA.123.323223 38166463

[68] Luo X Wang Y Zhu X Chen Y Xu B Bai X . MCL attenuates atherosclerosis by suppressing macrophage ferroptosis via targeting KEAP1/NRF2 interaction. Redox Biol. (2023) 69:102987. doi: 10.1016/j.redox.2023.102987 38100883 PMC10761782

[69] Liu Z Cheng S Zheng X Wang X Lu W Wang X . Paclitaxel attenuates atherosclerosis by suppressing macrophage ferroptosis and improving lipid metabolism via the Sirt1/Nrf2/GPX4 pathway. 39(15):e70917. doi: 10.1096/fj.202501047RR 40779351

[70] Tricetin attenuates atherosclerosis by suppressing macrophage ferroptosis via activation of the NRF2 pathway - ScienceDirect. Available online at: https://www.sciencedirect.com/science/article/abs/pii/S1567576924019404?via%3Dihub (Accessed May 11, 2026). 10.1016/j.intimp.2024.113418 39442188

[71] IRF5 siRNA nanoimmunotherapy: Restoring macrophage efferocytosis in atherosclerosis Circulation. Available online at: https://www.ahajournals.org/doi/10.1161/CIRCULATIONAHA.125.075352 (Accessed May 11, 2026). 10.1161/CIRCULATIONAHA.125.075352 41159271

[72] Ma C Hua Y Yang S Zhao Y Zhang W Miao Y . Wogonin attenuates atherosclerosis via KLF11-mediated suppression of PPARα-YAP1-driven glycolysis and enhancement of ABCA1/G1-mediated cholesterol efflux. Adv Sci. (2025) 12:2500610. doi: 10.1002/advs.202500610 40397286 PMC12199419

[73] Hansson GK Hermansson A . The immune system in atherosclerosis. Nat Immunol. (2011) 12:204–12. doi: 10.1038/ni.2001 21321594

[74] Kumar Y Hooda K Li S Goyal P Gupta N Adeb M . Abdominal aortic aneurysm: Pictorial review of common appearances and complications. Ann Transl Med. (2017) 5:256. doi: 10.21037/atm.2017.04.32 28706924 PMC5497081

[75] Dale MA Ruhlman MK Baxter BT . Inflammatory cell phenotypes in AAAs: Their role and potential as targets for therapy. Arterioscler Thromb Vasc Biol. (2015) 35:1746–55. doi: 10.1161/ATVBAHA.115.305269 26044582 PMC4514552

[76] Anagnostakos J Lal BK . Abdominal aortic aneurysms. Prog Cardiovasc Dis. (2021) 65:34–43. doi: 10.1016/j.pcad.2021.03.009 33831398

[77] Shimizu K Mitchell RN Libby P . Inflammation and cellular immune responses in abdominal aortic aneurysms. Arterioscler Thromb Vasc Biol. (2006). doi: 10.1161/01.ATV.0000214999.12921.4f 16497993

[78] Davis FM Tsoi LC Melvin WJ denDekker A Wasikowski R Joshi AD . Inhibition of macrophage histone demethylase JMJD3 protects against abdominal aortic aneurysms | journal of experimental medicine | rockefeller university press. 218(6):e20201839. doi: 10.1084/jem.20201839 PMC8008365 33779682

[79] Zhu J Meganathan I MacAruthur R Kassiri Z . Inflammation in abdominal aortic aneurysm: Cause or comorbidity? Can J Cardiol. (2024) 40:2378–91. doi: 10.1016/j.cjca.2024.08.274 39181326

[80] Hou N Zhou H Li J Xiong X Deng H Xiong S . Macrophage polarization and metabolic reprogramming in abdominal aortic aneurysm. 12(11):e1268. doi: 10.1002/iid3.1268 PMC11555488 39530309

[81] Bi C Liu B Gao P Wang C Fang S Huo Z . RAGE deficiency ameliorates abdominal aortic aneurysm progression. Inflammation Res. (2025) 74:63. doi: 10.1007/s00011-025-02027-2 40244438

[82] Huang J Jiang Y Ji R Jia Y Wang S Zhou Z . Macrophage scavenger receptor A1 antagonizes abdominal aortic aneurysm via upregulating IRG1. Biochem Pharmacol. (2023) 213:115631. doi: 10.1016/j.bcp.2023.115631 37257722

[83] Xu J Zhao J Chen H Tan X Zhang W Xia Z . Mesenchymal stromal cell-derived exosomes protect against abdominal aortic aneurysm formation through CD74 modulation of macrophage polarization in mice. Stem Cell Res Ther. (2024) 15:242. doi: 10.1186/s13287-024-03808-y 39098899 PMC11299418

[84] Wu Z Xu Z Pu H Ding A Hu J Lei J . NINJ1 facilitates abdominal aortic aneurysm formation via blocking TLR4-ANXA2 interaction and enhancing macrophage infiltration. 11(31):e2306237. doi: 10.1002/advs.202306237 PMC11336960 38922800

[85] Cai H Li H Xiao X Wang S Liu R Qin Y . TRAF6 promotes abdominal aortic aneurysm development by activating macrophage pyroptosis via the NLRP3/Caspase1/GSDMD pathway. FASEB J. (2025) 39:e70318. doi: 10.1096/fj.202402873R 39831511

[86] Xie C Ye F Zhang N Huang Y Pan Y Xie X . CCL7 contributes to angiotensin II-induced abdominal aortic aneurysm by promoting macrophage infiltration and pro-inflammatory phenotype. 25(15):7280–93. doi: 10.1111/jcmm.16757 PMC8335673 34189838

[87] Li Y Li R Li Y Li G Zhao Y Mou H . Transcription factor TCF3 promotes macrophage-mediated inflammation and MMP secretion in abdominal aortic aneurysm by regulating miR-143-5p/CCL20. J Cardiovasc Pharmacol. (2023) 82:458. doi: 10.1097/FJC.0000000000001484 37721971 PMC10691663

[88] Wu W Tang W Liang W Li Q Qi X Gao R . GDF15 suppresses abdominal aortic aneurysm by upregulating AREG expression to adjust macrophage polarization. Int Immunopharmacol. (2025) 159:114899. doi: 10.1016/j.intimp.2025.114899 40414071

[89] Lopez-Sanz L Bernal S Jimenez-Castilla L Prieto I Manna SL Gomez-Lopez S . Fcγ receptor activation mediates vascular inflammation and abdominal aortic aneurysm development. 11(7):e463. doi: 10.1002/ctm2.463 PMC8255062 34323424

[90] Cai D Sun C Murashita T Que X Chen S-Y . ADAR1 non-editing function in macrophage activation and abdominal aortic aneurysm. Circ Res. (2023) 132:e78–93. doi: 10.1161/CIRCRESAHA.122.321722 36688311 PMC10316962

[91] Peng Z Lv S-J Chen H Rao H Guo Z Wan Q . Disruption of PCSK9 suppresses inflammation and attenuates abdominal aortic aneurysm formation. Arterioscler Thromb Vasc Biol. (2025) 45(1):e1–14. doi: 10.1161/ATVBAHA.123.320391 39588646

[92] Wang Z Cheng J Wang Y Yuan H Bi S Wang S . Macrophage ILF3 promotes abdominal aortic aneurysm by inducing inflammatory imbalance in male mice. Nat Commun. (2024) 15:7249. doi: 10.1038/s41467-024-51030-4 39179537 PMC11344041

[93] Yu H Jiao X Lv Q Li L Du Y Hu C . ATF4 contributes to abdominal aortic aneurysm formation via modulating M1 macrophage polarization and inflammation. Aging Dis. (2024) 16:1691–708. doi: 10.14336/AD.2024.0116 38913045 PMC12101539

[94] Ma D Zheng B Suzuki T Zhang R Jiang C Bai D . Inhibition of KLF5–Myo9b–RhoA pathway–mediated podosome formation in macrophages ameliorates abdominal aortic aneurysm. Circ Res. (2017) 120(5):799–815. doi: 10.1161/CIRCRESAHA.116.310367 28115390

[95] Liu Z Fitzgerald M Meisinger T Batra R Suh M Greene H . CD95-ligand contributes to abdominal aortic aneurysm progression by modulating inflammation. 115(4):807–18. doi: 10.1093/cvr/cvy264 PMC6432056 30428004

[96] Olfr2 promotes recruitment of monocytes via CX3CR1 in abdominal aortic aneurysm | circulation research. (Accessed April 22, 2026). doi: 10.1161/CIRCRESAHA.125.326591 41473953

[97] Zhang Z Wu M Yao L Zhou W Liu X Chen Z . Trem2/tyrobp signaling protects against aortic dissection and rupture by inhibiting macrophage activation in mice. Arterioscler Thromb Vasc Biol. (2025) 45:119–35. doi: 10.1161/ATVBAHA.124.321429 39508103

[98] Cai D Li C Zhang Y He S Guo Y Liao W . CircHipk3 serves a dual role in macrophage pyroptosis by promoting NLRP3 transcription and inhibition of autophagy to induce abdominal aortic aneurysm formation. 14(12):e70102. doi: 10.1002/ctm2.70102 PMC11599875 39601144

[99] Mangum K Gallagher K Davis FM Mangum K Gallagher K Davis FM . The role of epigenetic modifications in abdominal aortic aneurysm pathogenesis. Biomolecules. (2022) 12. doi: 10.3390/biom12020172 35204673 PMC8961599

[100] Toczek J Boodagh P Sanzida N Ghim M Salarian M Gona K . Computed tomography imaging of macrophage phagocytic activity in abdominal aortic aneurysm. Theranostics. (2021) 11:5876–88. doi: 10.7150/thno.55106 33897887 PMC8058712

[101] Dang G Li T Yang D Yang G Du X Yang J . T lymphocyte-derived extracellular vesicles aggravate abdominal aortic aneurysm by promoting macrophage lipid peroxidation and migration via pyruvate kinase muscle isozyme 2. Redox Biol. (2022) 50:102257. doi: 10.1016/j.redox.2022.102257 35149342 PMC8842084

[102] Wagenhäuser MU Mulorz J Krott KJ Bosbach A Feige T Rhee YH . Crosstalk of platelets with macrophages and fibroblasts aggravates inflammation, aortic wall stiffening, and osteopontin release in abdominal aortic aneurysm. 10.1093/cvr/cvad168 37976180

[103] Luo Q Meng W Wang J Zhang X Meng Q Hu M . Macrophage-biomimetic nanomedicine for targeted therapy of abdominal aortic aneurysm via Nrf2/NF-κB pathway. Theranostics. (2026) 16:3363–83. doi: 10.7150/thno.123428 41608572 PMC12846727

[104] Xue C Jiang L Zhang B Sun J Zhu H Lu L . Integrative analysis reveals chemokines CCL2 and CXCL5 mediated shear stress-induced aortic dissection formation. Heliyon. (2024) 10:e23312. doi: 10.1016/j.heliyon.2023.e23312 38163105 PMC10757018

[105] Postnov A Suslov A SoBenin I Chairkin I Sukhorukov V Ekta MB . Thoracic aortic aneurysm: Blood pressure and inflammation as key factors in the development of aneurysm dissection Http://Www.Eurekaselect.Com. Available online at: https://www.eurekaselect.com/article/114093 (Accessed January 8, 2026). 10.2174/1381612827666210210142200 33568026

[106] Guo G Booms P Halushka M Dietz HC Ney A Stricker S . Induction of macrophage chemotaxis by aortic extracts of the mgR marfan mouse model and a GxxPG-containing fibrillin-1 fragment. Circulation. (2006) 114(17):1855–62. doi: 10.1161/CIRCULATIONAHA.105.601674 17030689

[107] Kimura K Motoyama E Kanki S Asano K Sips P Sheikh MAA . Novel aortic dissection model links endothelial dysfunction and immune infiltration. Circ Res. (2025) 137:26–42. doi: 10.1161/CIRCRESAHA.125.326230 40365676 PMC12175827

[108] Zhao Y-F Zuo Z-A Li Z-Y Yuan Y Hong S-C Fu W-G . Integrated multi-omics profiling reveals neutrophil extracellular traps potentiate aortic dissection progression. Nat Commun. (2024) 15:10736. doi: 10.1038/s41467-024-55038-8 39737994 PMC11686284

[109] Liu X Chen W Zhu G Yang H Li W Luo M . Single-cell RNA sequencing identifies an Il1rn+/Trem1+ macrophage subpopulation as a cellular target for mitigating the progression of thoracic aortic aneurysm and dissection. Cell Discov. (2022) 8:11. doi: 10.1038/s41421-021-00362-2 35132073 PMC8821555

[110] Li Y Ren P Dawson A Vasquez HG Ageedi W Zhang C . Single-cell transcriptome analysis reveals dynamic cell populations and differential gene expression patterns in control and aneurysmal human aortic tissue. Circulation. (2020) 142(14):1374–88. doi: 10.1161/CIRCULATIONAHA.120.046528 33017217 PMC7539140

[111] Luo M Zhao F Cheng H Su M Wang Y . Macrophage polarization: An important role in inflammatory diseases. Front Immunol. (2024) 15. doi: 10.3389/fimmu.2024.1352946 38660308 PMC11039887

[112] Cifani N Proietta M Tritapepe L Gioia D Ferri L Taurino M . Stanford-a acute aortic dissection, inflammation, and metalloproteinases: A review Ann Med (2015). Available online at: https://www.tandfonline.com/doi/abs/10.3109/07853890.2015.1073346 (Accessed January 7, 2026). 10.3109/07853890.2015.1073346 26339779

[113] Andreata F Syvannarath V Clement M Delbosc S Guedj K Fornasa G . Macrophage CD31 signaling in dissecting aortic aneurysm. J Am Coll Cardiol. (2018) 72:45–57. doi: 10.1016/j.jacc.2018.04.047 29957231

[114] Zhang Z Xie L Lin X He J Xie Y Li J . Ursodeoxycholic acid alleviates aortic aneurysm and dissection through the intestinal farnesoid X receptor/ceramide synthase 2 axis. Commun Biol. (2025) 8:1009. doi: 10.1038/s42003-025-08403-2 40617906 PMC12228684

[115] Chen X Chen R Wu Y Yu A Wang F Ying C . FABP5+ macrophages contribute to lipid metabolism dysregulation in type a aortic dissectionFABP5+. Int Immunopharmacol. (2024) 143:113438. doi: 10.1016/j.intimp.2024.113438 39447410

[116] Li S Liu G Cheng S Li X Weng X Yang J . Pharmacological and genetic inhibition of BTK ameliorates vascular degeneration, dissection, and rupture. Life Sci. (2025) 369:123533. doi: 10.1016/j.lfs.2025.123533 40049365

[117] Zhang K Li R Matniyaz Y Yu R Pan J Liu W . Liraglutide attenuates angiotensin II-induced aortic dissection and aortic aneurysm via inhibiting M1 macrophage polarization in APOE -/- mice. Biochem Pharmacol. (2024) 223:116170. doi: 10.1016/j.bcp.2024.116170 38548245

[118] Han T Tang H Lin C Yan D Zhou Z Yang Y . Costunolide mitigates inflammation and promotes extracellualr matrix integrity of thoracic aortic dissection by inhibiting NF-κB signaling. Int Immunopharmacol. (2024) 131:111784. doi: 10.1016/j.intimp.2024.111784 38493694

[119] Lian G Li X Zhang L Zhang Y Sun L Zhang X . Macrophage metabolic reprogramming aggravates aortic dissection through the HIF1α-ADAM17 pathway☆. eBioMedicine. (2019) 49:291–304. doi: 10.1016/j.ebiom.2019.09.041 31640947 PMC6945268

[120] Wang X Zhang H Ge Y Cao L He Y Sun G . AT1R regulates macrophage polarization through YAP and regulates aortic dissection incidence. Front Physiol. (2021) 12:644903. doi: 10.3389/fphys.2021.644903 34305627 PMC8299470

[121] Cui H Chen Y Li K Zhan R Zhao M Xu Y . Untargeted metabolomics identifies succinate as a biomarker and therapeutic target in aortic aneurysm and dissection. 42(42):4373–85. doi: 10.1093/eurheartj/ehab605 PMC11506060 34534287

[122] Tang M Wang M Wang Z Jiang B . RBM15 activates glycolysis in M1-type macrophages to promote the progression of aortic aneurysm and dissection. Int J Med Sci. (2024) 21:1976–89. doi: 10.7150/ijms.97185 39113895 PMC11302562

[123] Pan L Bai P Weng X Liu J Chen Y Chen S . Legumain is an endogenous modulator of integrin αvβ3 triggering vascular degeneration, dissection, and rupture. Circulation. (2022) 145:659–74. doi: 10.1161/CIRCULATIONAHA.121.056640 35100526

[124] ACKR1hiECs Promote Aortic Dissection Through Adjusting Macrophage Behavior | Circulation Research. (Accessed May 22, 2026). doi: 10.1161/CIRCRESAHA.124.325458 39692014

[125] Luo W Wang Y Zhang L Ren P Zhang C Li Y . Critical role of cytosolic DNA and its sensing adaptor STING in aortic degeneration, dissection, and rupture. Circulation. (2020) 141:42–66. doi: 10.1161/CIRCULATIONAHA.119.041460 31887080 PMC6939474

[126] Chakraborty A Li Y Zhang C Li Y Rebello KR Li S . Epigenetic induction of smooth muscle cell phenotypic alterations in aortic aneurysms and dissections. Circulation. (2023) 148:959–77. doi: 10.1161/CIRCULATIONAHA.123.063332 37555319 PMC10529114

[127] Teng X Wang Y Huang H Ding Y Wang J Liu M . Calpain-2-mediated endothelial focal adhesion disruption in thoracic aortic dissection. Adv Sci. (2025) 12:2501112. doi: 10.1002/advs.202501112 40171827 PMC12224997

[128] Aoki H Majima R Hashimoto Y Hirakata S Ohno-Urabe S . Ying and yang of Stat3 in pathogenesis of aortic dissection. J Cardiol. (2021) 77:471–4. doi: 10.1016/j.jjcc.2020.10.010 33148468

[129] Yin Z-Q Wen T Cao X-L Lv Z-X Su Y Liang L . The transcription factor RBPJ is required for inflammatory macrophage activation in thoracic aortic dissection by mediating mechanotransduction-induced glycolysis. Cell Mol Life Sci. (2025) 82:370. doi: 10.1007/s00018-025-05908-1 41148245 PMC12569315

[130] Wang A Dong S Liu B Liu D Zou M Han Y . The role of RUNX1/NF-κB in regulating PVAT inflammation in aortic dissection. Sci Rep. (2024) 14:9960. doi: 10.1038/s41598-024-60737-9 38693222 PMC11063189

[131] Li Y Yu J Chen W Wang X Tan X Cui J . 12-HETE is an endogenous modulator of BLT2 triggering vascular degeneration, dissection, and rupture. Adv Sci. (2025) 13:e15897. doi: 10.1002/advs.202515897 41194414 PMC12849872

[132] Zhang Y Qu H Li C Li L He L . The S-nitrosylation of Septin2 (SNO-Septin2) axis: A novel potential therapeutic target for treating aneurysms and dissection. Drug Discov Ther. (2024) 18:207–9. doi: 10.5582/ddt.2024.01047 38987209

[133] Zhang L Zhou J Jing Z Xiao Y Sun Y Wu Y . Glucocorticoids regulate the vascular remodeling of aortic dissection via the p38 MAPK-HSP27 pathway mediated by soluble TNF-RII. eBioMedicine. (2018) 27:247–57. doi: 10.1016/j.ebiom.2017.12.002 29287621 PMC5828293

[134] Yuan Z Chen R Fan R Yang Z Zhu Q Qiu C . FOSL2+ macrophages drive pro-inflammatory phenotype via miR-92a-3p/FOSL2/NF-κB axis to mediate endothelial cell injury in aortic dissectionFOSL2+. Life Sci. (2025), 388:124174. doi: 10.1016/j.lfs.2025.124174 41453656

[135] Al-Rifai R Vandestienne M Lavillegrand J-R Mirault T Cornebise J Poisson J . JAK2V617F mutation drives vascular resident macrophages toward a pathogenic phenotype and promotes dissecting aortic aneurysm. Nat Commun. (2022) 13:6592. doi: 10.1038/s41467-022-34469-1 36329047 PMC9633755

[136] Liu Y Zou L Tang H Li J Liu H Jiang X . Single-cell sequencing of immune cells in human aortic dissection tissue provides insights into immune cell heterogeneity. Front Cardiovasc Med. (2022) 9:791875. doi: 10.3389/fcvm.2022.791875 35433892 PMC9008490

[137] He W Yu S Li J Li S Chen Z Zhang J . From inflammation to remodelling: A novel BASP1+ monocyte subset as a catalyst for acute aortic dissection. J Adv Res. (2025) 78:647–66. doi: 10.1016/j.jare.2025.03.003 40057028 PMC12684931

[138] Ungvari Z Tarantini S Donato AJ Galvan V Csiszar A . Mechanisms of vascular aging. Circ Res. (2018) 123:849–67. doi: 10.1161/CIRCRESAHA.118.311378 30355080 PMC6248882

[139] Vendrov AE Lozhkin A Hayami T Levin J Silveira Fernandes Chamon J Abdel-Latif A . Mitochondrial dysfunction and metabolic reprogramming induce macrophage pro-inflammatory phenotype switch and atherosclerosis progression in aging. Front Immunol. (2024) 15:1410832. doi: 10.3389/fimmu.2024.1410832 38975335 PMC11224442

[140] The NLRP3 Inflammasome as a Critical Actor in the Inflammaging Process. Available online at: https://www.mdpi.com/2073-4409/9/6/1552 (Accessed May 22, 2026).

[141] Meyers AK Zhu X . The NLRP3 inflammasome: Metabolic regulation and contribution to inflammaging. Cells. (2020) 9:1808. doi: 10.3390/cells9081808 32751530 PMC7463618

[142] Tyrrell DJ Goldstein DR . Ageing and atherosclerosis: Vascular intrinsic and extrinsic factors and potential role of IL-6. Nat Rev Cardiol. (2021) 18:58–68. doi: 10.1038/s41569-020-0431-7 32918047 PMC7484613

[143] Covarrubias AJ Aksoylar HI Yu J Snyder NW Worth AJ Iyer SS . Akt-mTORC1 signaling regulates acly to integrate metabolic input to control of macrophage activation. eLife. (2016). 5:e11612. doi: 10.7554/eLife.11612 26894960 PMC4769166

[144] Covarrubias AJ Perrone R Grozio A Verdin E . NAD+ metabolism and its roles in cellular processes during ageing. Nat Rev Mol Cell Biol. (2021) 22:119–41. doi: 10.1038/s41580-020-00313-x 33353981 PMC7963035

[145] Yarbro JR Emmons RS Pence BD . Macrophage immunometabolism and inflammaging: Roles of mitochondrial dysfunction, cellular senescence, CD38, and NAD. Immunometabolism. (2020) 2:e200026. doi: 10.20900/immunometab20200026 32774895 PMC7409778

[146] Rodriguez Morales D Larcher V Ruz Jurado M Arifaj D Tombor LS Zanders L . Vascular niches are the primary hotspots in cardiac aging. Circ Res. (2025) 137:1353–67. doi: 10.1161/CIRCRESAHA.125.327060 41090219 PMC12591552

[147] Lee H-Y Kim H-K Hoang T-H Yang S Kim H-R Chae H-J . The correlation of IRE1α oxidation with Nox4 activation in aging-associated vascular dysfunction. Redox Biol. (2020) 37:101727. doi: 10.1016/j.redox.2020.101727 33010578 PMC7530295

[148] Liberale L Badimon L Montecucco F Lüscher TF Libby P Camici GG . Inflammation, aging and cardiovascular disease: JACC review topic of the week. J Am Coll Cardiol. (2022) 79:837–47. doi: 10.1016/j.jacc.2021.12.017 35210039 PMC8881676

[149] Weinberger T Esfandyari D Messerer D Percin G Schleifer C Thaler R . Ontogeny of arterial macrophages defines their functions in homeostasis and inflammation. Nat Commun. (2020) 11:4549. doi: 10.1038/s41467-020-18287-x 32917889 PMC7486394

[150] Blériot C Chakarov S Ginhoux F . Determinants of resident tissue macrophage identity and function. Immunity. (2020) 52:957–70. doi: 10.1016/j.immuni.2020.05.014 32553181

[151] The Role of Macrophages in Vascular Repair and Regeneration After Ischemic Injury. Available online at: https://www.mdpi.com/1422-0067/21/17/6328 (Accessed May 22, 2026). 10.3390/ijms21176328 PMC7503238 32878297

[152] Establishment and Maintenance of the Macrophage Niche: Immunity. Available online at: https://www.cell.com/immunity/fulltext/S1074-7613(20)30086-8?_returnURL=https%3A%2F%2Flinkinghub.elsevier.com%2Fretrieve%2Fpii%2FS1074761320300868%3Fshowall%3Dtrue (Accessed May 22, 2026). 10.1016/j.immuni.2020.02.015 32187515

[153] Cautivo KM Steer CA Molofsky AB . Immune outposts in the adventitia: One foot in sea and one on shore. Curr Opin Immunol. (2020) 64:34–41. doi: 10.1016/j.coi.2020.03.005 32339862 PMC7584777

